# Identifying 20 homogeneous clusters of acute patients discharged with nonspecific diagnoses through k-prototypes mixed data clustering

**DOI:** 10.1186/s12873-025-01459-7

**Published:** 2026-01-10

**Authors:** Rasmus Gregersen Mottlau, Marie Villumsen, Axel Nyström, Hanne Nygaard, Jens Rasmussen, Mikkel B. Christensen, Jakob Lundager Forberg, Janne Petersen

**Affiliations:** 1 Department of Emergency Medicine, Copenhagen University Hospital – Bispebjerg and Frederiksberg, Copenhagen, NV 2400 Denmark; 2 Center for Clinical Research and Prevention, Copenhagen University Hospital – Bispebjerg and Frederiksberg, Copenhagen, Denmark; 3 Department of Public Health, Faculty of Health and Medical Sciences, University of Copenhagen, Copenhagen, Denmark; 4 Department of Laboratory Medicine, Lund University, Lund, Sweden; 5 Copenhagen Center for Translational Research, Copenhagen University Hospital – Bispebjerg and Frederiksberg, Copenhagen, Denmark; 6 Department of Clinical Pharmacology, Copenhagen University Hospital – Bispebjerg and Frederiksberg, Copenhagen, Denmark; 7 Department of Clinical Medicine, University of Copenhagen, Copenhagen, Denmark; 8 Department of Emergency Medicine, Helsingborg Hospital, Helsingborg, Sweden

**Keywords:** Acute medicine, Clustering, Emergency medicine, Nonspecific diagnoses, Noncausative diagnosis, Unspecific diagnoses, Unsupervised machine learning

## Abstract

**Background:**

Patients discharged with nonspecific diagnoses after acute hospital care are frequent and represent potential diagnostic uncertainty at discharge. Adverse outcomes indicate missed diagnoses with a potential for improving patient safety. However, research and interventions are limited by population heterogeneity. We aimed to identify clusters of patients discharged with nonspecific diagnoses by employing unsupervised machine learning and to assess the risk of readmission and mortality of each cluster.

**Methods:**

Observational, register-based study of emergency department arrivals discharged with nonspecific diagnoses (ICD-10: R and Z03 chapters) from March 2019 to February 2020 in Denmark. We applied partitional (k-prototypes) and hierarchical (agglomerative) clustering based on demographics, socioeconomics, comorbidities, administrative information, biochemistry, and 50 nonspecific discharge diagnosis groups. The risk of 30-day readmission and mortality after discharge was assessed as cumulative incidence for each cluster.

**Results:**

We included 92,650 patients. A 20 clusters k-prototypes model best fitted our data. Clusters 1–5 were differentiated by no or limited biochemistry across different age and comorbidity patterns. Clusters 6–9 consisted mainly of young adults with low comorbidity, except Cluster 9 with notable neuropsychiatric and substance abuse comorbidities. Clusters 10–20 described the older patients: 10–14 with single comorbidities and 15–20 with substantial comorbidity of different cooccurring patterns. The risk of 30-day readmission and mortality ranged from 5% to 27% and 0% to 9% across clusters, respectively.

**Conclusion:**

Patients with nonspecific discharge diagnoses after acute hospital contacts can be grouped into 20 distinct clusters based on clinical, socioeconomic, administrative, and biochemical features. The clusters can be used to form delimited populations allowing for better and more individualized prediction models.

**Supplementary Information:**

The online version contains supplementary material available at 10.1186/s12873-025-01459-7.

## Introduction

The main purposes of EDs includes identifying and treating the critically ill and to rule out time-critical conditions in undifferentiated patients. If a time-critical conditions is not suspected and the patient appear fit for discharge, they can be discharged even though a cause of the complaints has not been established. These patients should be registered with a nonspecific diagnosis, which constitute a large proportion of Emergency Department (ED) non-trauma patients, at 26–51% [1, 2]. Despite the absence of any time-critical diagnosis during their ED or hospital stay, these patients have been reported to have a notable risk of adverse outcomes [2–9], which suggest a degree of diagnostic errors [10]. A better understanding of the population could therefore lead to better identification high risk patients and missed diagnoses leading to improved patient care. Despite the substantial number of patients with nonspecific discharge diagnoses and their non-negligible short-term risk, research on this group remains limited.

Nonspecific diagnoses are numerous and heterogeneous. In a previous work, we reduced the 337 registered nonspecific discharge diagnoses (ICD-10) from acutely attending patients into 50 clinical subgroups by audit of the diagnose codes [2]. These subgroups exhibited considerable variation in patient characteristics. Such differences present challenges for application of stratification and prediction models. It remains unknown if we can form fewer and more homogeneous subgroups by combining these clinical subgroups with other patient characteristics. Therefore, new methods for categorizing patients with nonspecific diagnoses are warranted.

A novel approach for subgrouping patients discharged with nonspecific diagnoses is clustering based on unsupervised machine learning, which has shown promising results in other acute care domains [11–17]. These techniques form clusters where observations within clusters are similar but also dissimilar to observations in the other clusters based on the included information [18–21]. Applying unsupervised machine learning to the heterogeneous population of patients discharged with nonspecific discharge diagnoses could yield new insight into the structure of the data and reveal clinically meaningful subgroups. These subgroups could facilitate better and more targeted research and interventions. For instance, biomarkers or risk factors could display a higher importance for a homogeneous subgroup, that would otherwise be masked in a larger, heterogeneous population. Better predictive accuracy has also been achieved by combining unsupervised and supervised machine learning in a so-called cluster-then-predict approach [22]. 

The aim of the study was to identify homogeneous and clinically distinct clusters of patients discharged with nonspecific diagnoses after ED hospital care episodes by employing unsupervised machine learning methods. Further, we aimed to describe the prognosis and characteristics of each cluster.

## Methods

### Study design, setting, and data sources

We conducted an observational, register-based study across four out of five regions of Denmark during the study period from March 1, 2019, to February 28, 2020. We utilized an existing database at Statistics Denmark [23], combining information from the Danish National Patient Register [24], the Register of Laboratory Results for Research [25], the Central Person Register [26], the Danish Education Register [27], and the Income Statistics Register [28]. Data from the Central Denmark Region was omitted due to missing information on laboratory results. Information about the Danish, tax-funded, universal healthcare structure has been described in detail elsewhere [23, 29, 30]. Acute hospital care enforces mandatory referral through general practitioners, out-of-hours doctors, emergency medical systems, or telephonic medical helplines, although self-referrals will be treated if needing urgent care. Further, a transition towards receiving all types of acute patients in EDs has mostly finished.

### Study population

For inclusion, we considered hospital care episodes beginning in EDs where the patient was discharged with a primary nonspecific diagnosis from the R and Z03 ICD-10 chapters either directly from the ED without admission or after in-hospital stay (admissions). The few diagnoses from the R and Z03 ICD-10 chapters that we considered disease-specific were excluded (Table S1) [2]. In the Capital Region, out-of-hours consultations are registered as ED visits in the DNPR [23, 30]. To exclude these and standardize the data across the regions we excluded hospital care episodes lasting less than 3 h. We excluded total hospital stays longer than 168 h (7 days) to avoid nonspecific discharge diagnoses that likely indicate misregistration rather than unresolved issues. We excluded several groups of patients (Table S2). First, those who terminated further diagnostics by their own choice (left against medical advice). Second patients with a registered pregnancy as they present markedly different possible symptoms and conditions along with diagnostic considerations for the fetus, altering the diagnostic process. Third, patients discharged from psychiatric departments as they indicate a primary psychiatric condition rather than somatic symptoms. Fourth, patients in known palliative care as they could have been discharged with the purpose of dying at home, opting out of further diagnostics resulting in a nonspecific diagnosis. Finally, patients dying during the stay or the same day as discharge as it was not possible to clearly differentiate whether they died during stay or not. As full data was needed for the models, we also excluded patients with missing sociodemographic information, which largely included patients without Danish CPR-numbers (mainly tourists and illegal immigrants) necessary for follow-up. Patients were only included at first eligible contact.

Hospital care episodes were formed by combining contacts separated by less than 4 h using the %DNPR_contact_combine SAS Macro [31]. 

### Characteristics of the data

The patients were described and clustered based on demographics, socioeconomics, discharge diagnosis groups, comorbidities, administrative information, and biochemistry results (see Table 1 for included variables and Table S3 for socioeconomic and administrative variable definitions). We considered 50 grouped primary discharge diagnoses in concordance with our previous publication, seeking to combine symptoms describing similar symptoms [2]. For this present study, we reclassified groups with < 0.5% prevalence as “Other nonspecific diagnoses” (18 of 50 groups). Comorbidities were defined from the MultiMorbidity Measure (M3) index with a 10-year lookback period—we included both the total M3 score and single comorbidities, with prevalence ≥ 1.0% (13 of 61 comorbidities excluded; see Stanley (2017) for definitions) [32]. Biochemical results were evaluated during the hospital care episode and up to 8 h prior. If more than one of each biochemical result was available, only the latest was considered. We categorized these results as either normal, abnormal, or missing according to the laboratory reference values.

**Table 1:** Variables included for clustering

**Demographics** - Age - Sex	**Discharge diagnosis groups** (as one variable with multiple levels): - Headache - Observation for stroke - Fainting - Observation for nervous system disorder, unspecified - Seizures or muscle cramps - Symptoms involving the nervous and musculoskeletal system - Vertigo - Observation for concussion - Epistaxis and oropharyngeal bleeding - Chest pain - Observation for myocardial infarction - Palpitations - Abnormal heart rhythm - Observation for arrhythmia - Observation for other cardiovascular disease - Coughing - Abnormal breathing - Abdominal pain - Nausea and vomiting - Dysphagia and eating difficulties - Altered mental status and amnesia - Edema - Fever - Malaise or fatigue - Tendency to fall - Abnormal findings of the skin - Symptom from urination and urinary tract - Acute pain - Observation for unspecified disease or condition - Abnormal biochemical result - Observation for cancer - Other nonspecific diagnoses	**Comorbidities** - Total M3 score - Individual comorbidities: o Cerebrovascular disease o Mental and behavioral disorders due to brain damage o Other neurological disorders, excluding epilepsy o Myocardial infarction o Angina o Congestive heart failure o Cardiac arrhythmia o Cardiac valve disease o Cardiac disease (other) o Hypertension, uncomplicated o Peripheral vascular disease o Aortic and other aneurysms o Pulmonary circulation disorder o Chronic pulmonary disease o Diabetes (uncomplicated) o Diabetes (complicated) o Endocrine disorders o Metabolic disorder o Anemia deficiency o Coagulopathy and other blood disorders o Chronic renal o Gastrointestinal ulcer or upper GI disease o Inflammatory bowel disease o Intestinal disorder o Liver disease (moderate or severe) o Pancreatitis o Muscular peripheral nerve disorder o Joint or spinal disorder o Bone disorder o Osteoporosis (uncomplicated) o Connective tissue disease o Obesity o Malnutrition or other nutritional disorders o Urinary tract problem (chronic) o Inner ear disorder o Eye problem long term o Major psychiatric disease o Anxiety and behavioral disorders o Alcohol abuse o Drug abuse o Sleep disorder o Dementia o Colorectal cancer o Breast cancer o Prostate cancer o Other cancers o Metastatic cancers
**Socioeconomics** - Cohabitation - Educational level - Source of income (socioeconomic classification) - Marital status (civil status) - Origin		
**Administrative** - Length of stay - Time and day, start of contact - Time and day, end of contact		
**Biochemistry** - Haemoglobin - Leukocytes - Platelets - CRP - eGFR - Sodium - Potassium - Alanine transaminase (ALAT) - Alkaline phosphatase (ALP) - Bilirubin - Coagulation, measured as international normalised ratio (INR) - Glucose - Tropinin I/T - D-dimer		

### Outcomes

We assessed the accumulated 30-day risk of readmission and mortality after discharge as the patient’s prognosis. To focus on readmissions potentially involving missed diagnoses, a temporal criterion was applied in the definition: A readmission was defined as a new hospital care episode initiated acutely with a total length of stay of at least 12 h.

### Statistics, data processing, clustering model development and outcome analyses

Variables (Table 1) were presented as mean and standard deviation (SD) for numerical variables and frequency and proportion for categorical variables. In data preprocessing, continuous variables (age and total M3 comorbidity score) were normalized with the Yeo-Johnson power transformer [33]. Ordinal variables (educational level and categorized length of stay) were also treated as continuous variables. In total, we included four continuous variables and 69 categorical variables (Table 1).

We tested both partitional clustering (the k-prototypes algorithm, an extension of k-means to include categorical variables) and hierarchical clustering (agglomerative clustering) [34, 35]—please see the supportive methods section in the supplementary material. To test model stability and validity, we split the data into a train set (*n* = 50,000) and test set and conducted model visualizations separately (Figure S1–S3). As the models with a low number of clusters mainly differentiated clusters on age and associated comorbidities, we decided that this was not sufficient for identifying clinically distinct and relevant clusters of patients per our aim. Therefore, we only considered models of at least 6 clusters. We chose suitable model candidates based on visualizations of model statistics across the models of different number of clusters. Both the scree plot elbow points for partitional clustering and distances between cluster merges for hierarchical clustering, suggested final model candidates in the ranges 11–13 and 19–22 with no clear superior model (Figure S1–S3). The Silhouette scores were poor across these models ranging from 0.03 to 0.07. To choose the model best describing homogeneous and clinically distinct clusters, we chose the final model by evaluating the clusters identified by the models. This evaluation was based on an assessment of heatmaps and histograms by a team of clinicians and epidemiologists (co-authors RGM, MV, MBC, HN, JR, JF, and JP). Disagreements were settled by discussion until all assessors agreed. We based these evaluations on basic characteristics, histograms, and heatmaps showing the logarithmic relative prevalence of all variables within a cluster compared to the overall prevalence of the variables (Figure S4–S8). However, identifying superior models based on 73 variables proved difficult. Consequently, we used the inherent structure of hierarchical clustering, where each cluster is formed by merging two other sub-clusters iteratively. This approach allowed us to assess whether the sub-clusters were sufficiently distinct from each other and other existing clusters in the model. Beyond more than 20 clusters, the new clusters only differentiated on few parameters that were meaningful or distinct, whereas the 20 clusters were deemed clinically distinct. Therefore, we chose 20 clusters as the best hierarchical model. This model was then compared to a 20-cluster k-prototypes model, which was also among the final model candidates. Ultimately, we ended favoring the k-prototypes model, selecting it as the final model.

**Fig. 1:**
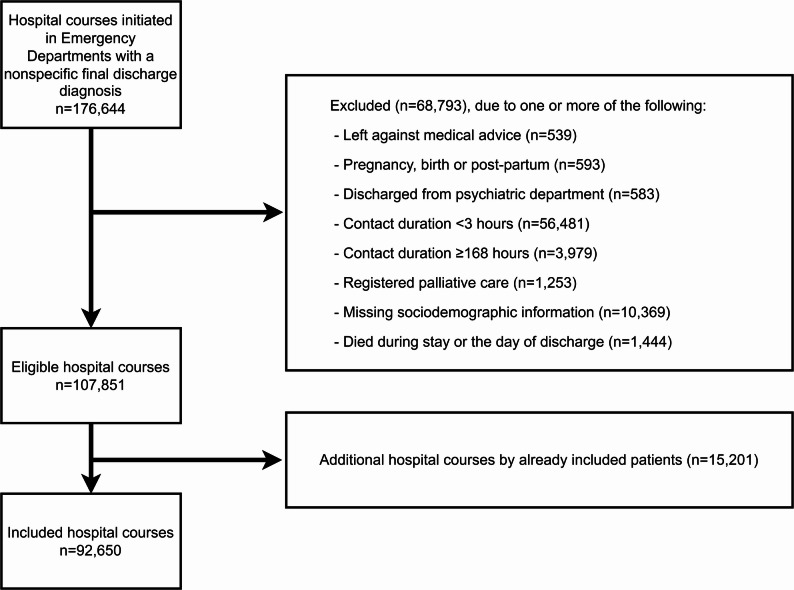
Inclusion and exclusion flow-chart. Note that patients can fulfill more than one exclusion criteria

The absolute risk of readmission and death was calculated as the cumulative incidence with 95% confidence interval (95% CI) as pointwise confidence limits by normal approximation. For analyses of readmission, death was modelled as a competing risk. Cluster were compared based on 95% CI.

To assess model stability, we produced and compared heat maps and outcomes measures for the train and test data separately.

Initial data management was done using SAS Enterprise Guide (version 8.3). Data preprocessing and clustering were done in Spyder (Python 3.9) using the sklearn, SciPy, and kmodes packages.

## Results

### Cohort

Over the 12-month period, we identified 176,644 patients presenting in EDs who were discharged with a nonspecific discharge diagnosis. After exclusion of 83,994 presentations, we included a total of 92,650 patients (Fig. 1). The cohort had a mean age of 58.1 (SD: 20.6), were mostly female (53.1%) and had a low comorbidity burden (median M3 score (Q1;Q3): 0.3 (0.0;1.0). The majority (*n* = 58,522 (63.2%)) were discharged within 12 h of arrival.

We used a random 50,000-sample to develop the model and compared model development visualizations with the remaining 42,650 patients as test data (Figure S1–S3). The test and train data yielded comparable model visualizations. The 20-clusters k-prototypes model was elected as the final model, as it had an increased differentiation of the older patients but lesser of the younger (fewer and larger clusters) compared to the hierarchical model.

### Cluster characteristics

**Fig. 2:**
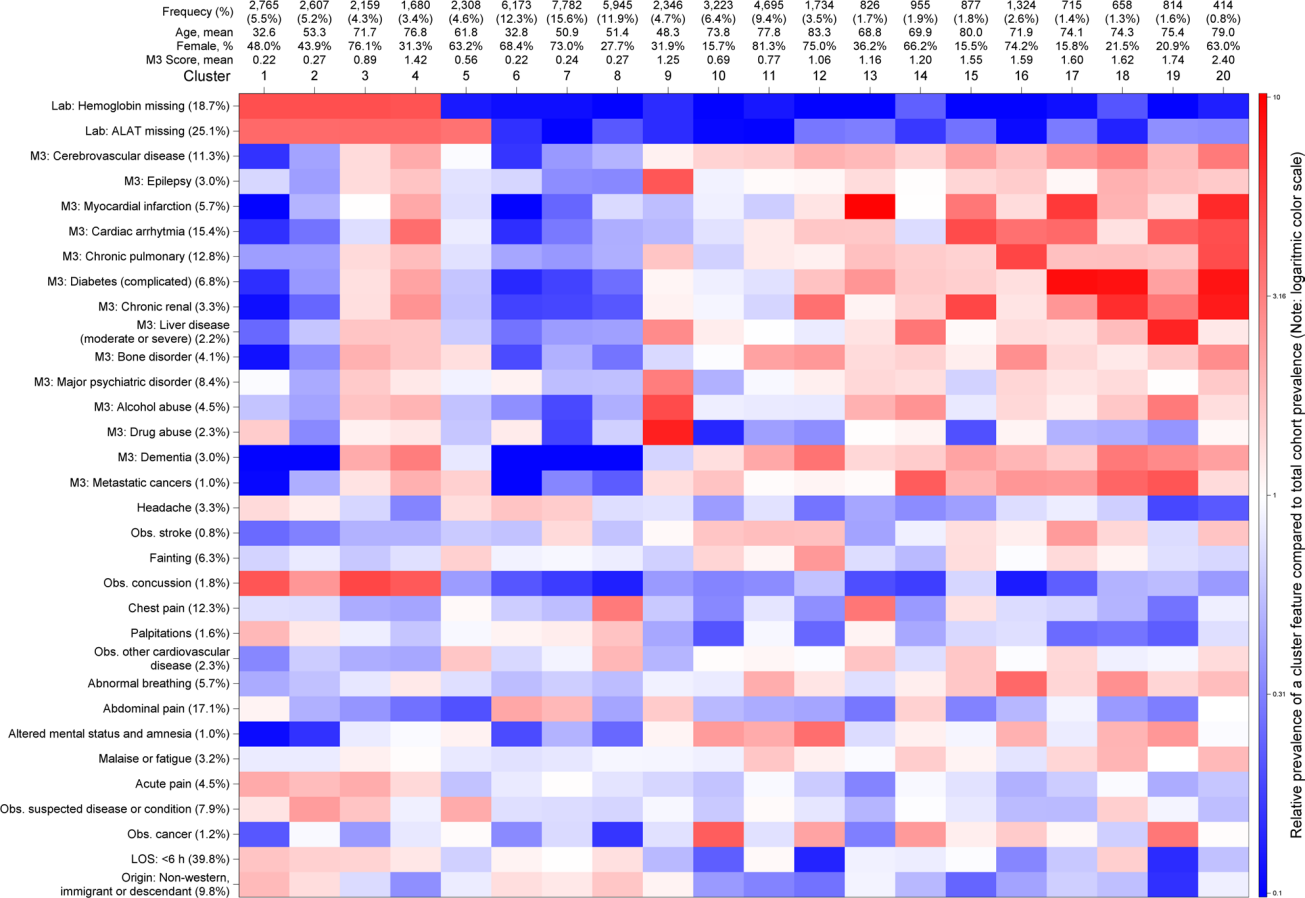
Heatmap of the relative prevalence of selected features (rows) in a cluster (columns) compared to the entire cohort prevalence. The color intensity represents lower (blue), similar (white) and elevated (red) relative prevalence using a logarithmic color scale

We identified an overall structure of the 20 final clusters: Clusters 1–5 shared patients with no or selected biochemistry, Clusters 6–9 grouped primarily young patients, and Clusters 10–20 grouped primarily older patients, comprising 23.0%, 44.5%, and 32.5% of the entire cohort, respectively. The clusters varied in size from 414 (0.8%) in Cluster 20 to 7,782 (15.6%) in Cluster 7. None of the nonspecific diagnosis groups were confined to one or a few clusters but were present across multiple clusters. Sociodemographic differences across clusters have been presented in Table 2, showing mean age 32.6–83.3, mean M3 score 0.22–2.40, female sex distribution of 16–81%, and high educational level 2–50%. Key features with high relative prevalence in a cluster compared to the entire cohort have been presented in Table 3. Similarly, Fig. 2 shows the prevalence of selected features in a cluster compared to the entire cohort as a heatmap of selected features (see Figure S4–S8 for heatmaps of all features). The test dataset had small variation in the overall prevalence of features but revealed very similar heat maps visualization indicating high reproducibility of the final model (Supplementary S9).

**Table 2:** Patient characteristics and cluster summary for the 20 clusters

Cluster	Frequency (%)	Demographics		Comorbidity	Prognosis	
		Age, mean (SD)	Female sex, %	M3 score, mean (SD)	Readmission, % (95% CI)	Mortality, % (95% CI
1	2,765 (5.5%)	32.6 (12.2)	48%	0.22 (0.40)	4.5% (3.8–5.3%)	Censored, few events
2	2,607 (5.2%)	53.3 (15.5)	44%	0.27 (0.43)	5.4% (4.8–6.1%)	0.3% (0.2–0.7%)
3	2,159 (4.3%)	71.7 (14.2)	76%	0.89 (0.82)	12% (11–14%)	2.1% (1.6–2.9%)
4	1,680 (3.4%)	76.8 (11.2)	31%	1.42 (0.94)	18% (16–19%)	2.7% (2.0-3.7%)
5	2,308 (4.6%)	61.8 (16.6)	63%	0.56 (0.68)	8.8% (7.8–10%)	0.6% (0.4–1.1%)
6	6,173 (12.3%)	32.8 (12.3)	68%	0.22 (0.36)	6.4% (5.8-7.0%)	Censored, few events
7	7,782 (15.6%)	50.9 (13.9)	73%	0.24 (0.39)	5.9% (5.4–6.4%)	0.1% (< 0.1–0.2%)
8	5,945 (11.9%)	51.4 (15.2)	28%	0.27 (0.42)	4.4% (3.9–4.8%)	Censored, few events
9	2,346 (4.7%)	48.3 (13.3)	32%	1.25 (0.88)	15% (14–17%)	0.8% (0.5–1.2%)
10	3,223 (6.4%)	73.8 (9.53)	16%	0.69 (0.74)	15% (14–16%)	2.2% (1.8–2.8%)
11	4,695 (9.4%)	77.8 (9.31)	81%	0.77 (0.74)	11% (10–12%)	1.2% (0.9–1.6%)
12	1,734 (3.5%3)	83.3 (8.77)	75%	1.06 (0.84)	19% (17–21%)	5.3% (4.5–6.2%)
13	826 (1.7%)	68.8 (11.3)	36%	1.16 (0.86)	11% (9.3–14%)	Censored, few events
14	955 (1.9%)	69.9 (12.7)	66%	1.20 (0.96)	26% (23–28%)	7.4% (6.1-9.0%)
15	877 (1.8%)	80.0 (7.92)	16%	1.55 (0.87)	18% (16–20%)	2.8% (2.0-4.1%)
16	1,324 (2.6%)	71.9 (10.8)	74%	1.59 (0.88)	17% (15–19%)	2.9% (2.2–3.9%)
17	715 (1.4%)	74.1 (8.99)	16%	1.60 (0.97)	15% (13–18%)	2.4% (1.5–3.7%)
18	658 (1.3%)	74.3 (10.3)	22%	1.62 (0.94)	16% (14–19%)	3.6% (2.4–5.6%)
19	814 (1.6%)	75.4 (11.1)	21%	1.74 (1.00)	27% (24–30%)	8.9% (7.1–11.3%)
20	414 (0.8%)	79.0 (9.73)	63%	2.40 (0.95)	24% (20–29%)	2.9% (1.7-5.0%)

95% confidence interval, calculated as pointwise confidence limits by normal approximation. Cells with < 5 events censored due to rules of micro-data protection

Abbreviations: 95% CI = 95% confidence interval

**Table 3:** Cluster key features – top 5 comorbidities and primary nonspecific discharge diagnosis group of increased relative prevalence in each cluster compared to the entire cohort

Cluster	Comorbidities (prevalence in entire cohort)	Prevalence in cluster	Primary discharge diagnosis group (prevalence in the entire cohort)	Prevalence in cluster
1	M3: Anxiety and behavioral disorders (6.9%)	11%	Obs. Concussion (1.8%)	8.6%
	M3: Drug abuse (2.3%)	3.6%	Acute pain (4.5%)	10%
			Abnormal findings of the skin (0.9%)	1.7%
			Palpitations (1.6%)	3.1%
2	None		Obs. Concussion (1.8%)	4.8%
			Epistaxis and oropharyngeal bleeding (1.7%)	4.4%
			Obs. Suspected disease or condition (7.9%)	19%
			Acute pain (4.5%)	8.2%
			Abnormal findings of the skin (0.9%)	1.5%
3	M3: Dementia (3.0%)	6.3%	Obs. Concussion (1.8%)	10%
	M3: Mental and behavioral disorders due to brain damage (1.4%)	2.8%	Epistaxis and oropharyngeal bleeding (1.7%)	9.1%
	M3: Bone disorder (4.1%)	8.4%	Dysphagia and eating difficulties (0.5%)	1.8%
	M3: Osteoporosis (uncomplicated) (3.0%)	5.7%	Acute pain (4.5%)	10%
	M3: Breast cancer (1.7%)	3.1%	Obs. Suspected disease or condition (7.9%)	13%
4	M3: Cardiac arrhythmia (15.4%)	58%	Obs. Concussion (1.8%)	8.3%
	M3: Congestive heart failure (6.0%)	20%	Dysphagia and eating difficulties (0.5%)	1.4%
	M3: Dementia (3.0%)	10%	Symptom from urination and urinary tract (3.3%)	8.1%
	M3: Prostate cancer (1.9%)	5.8%	Tendency to fall (1.3%)	2.7%
	M3: Cardiac valve (4.9%)	14%	Edema (0.9%)	1.4%
5	M3: Metastatic cancers (1.0%)	1.6%	Obs. Myocardial infarction (3.4%)	10%
	M3: Urinary tract problem (chronic) (1.3%)	1.8%	Obs. Suspected disease or condition (7.9%)	17%
	M3: Osteoporosis (uncomplicated) (3.0%)	4.1%	Symptoms involving the nervous and musculoskeletal systems (2.9%)	5.2%
	M3: Bone disorder (4.1%)	5.5%	Obs. Other cardiovascular disease (2.3%)	3.8%
	M3: Breast cancer (1.7%)	2.2%	Obs. Arrhythmia (0.6%)	1.0%
6	M3: Anxiety and behavioral disorders (6.9%)	12%	Abdominal pain (17.1%)	38%
	M3: Drug abuse (2.3%)	2.7%	Headache (3.3%)	5.6%
	M3: Major psychiatric disorder (8.4%)	9.4%	Seizures or muscle cramps (1.7%)	2.3%
	M3: Obesity (8.7%)	9.1%	Symptoms involving the nervous and musculoskeletal systems (2.9%)	4.0%
			Palpitations (1.6%)	1.8%
7	M3: Obesity (8.7%)	10%	Abdominal pain (17.1%)	33%
			Obs. Nervous system disorder, unspecified (1.2%)	2.0%
			Headache (3.3%)	5.2%
			Symptoms involving the nervous and musculoskeletal systems (2.9%)	4.5%
			Obs. Stroke (0.8%)	1.2%
8	None		Chest pain (12.3%)	41%
			Obs. Myocardial infarction (3.4%)	6.7%
			Obs. Other cardiovascular disease (2.3%)	4.3%
			Palpitations (1.6%)	2.8%
			Coughing (0.9%)	1.0%
9	M3: Drug abuse (2.3%)	17%	Seizures or muscle cramps (1.7%)	7.1%
	M3: Alcohol abuse (4.5%)	23%	Abnormal biochemical result (0.6%)	1.5%
	M3: Epilepsy (3.0%)	14%	Nausea and vomiting (1.5%)	2.7%
	M3: Mental and behavioral disorders due to brain damage (1.4%)	4.7%	Fever (1.5%)	2.6%
	M3: Anxiety and behavioral disorders (6.9%)	23%	Abdominal pain (17.1%)	27%
10	M3: Prostate cancer (1.9%)	7.1%	Obs. Cancer (1.2%)	5.2%
	M3: Colorectal cancer (1.3%)	2.5%	Symptom from urination and urinary tract (3.3%)	14%
	M3: Aortic and other aneurysms (1.9%)	3.5%	Fever (1.5%)	4.1%
	M3: Other cancers (1.9%)	3.3%	Altered mental status and amnesia (1.0%)	2.5%
	M3: Other neurological disorders (2.4%)	4.3%	Tendency to fall (1.3%)	2.7%
11	M3: Osteoporosis (uncomplicated) (3.0%)	7.6%	Tendency to fall (1.3%)	3.0%
	M3: Breast cancer (1.7%)	4.2%	Altered mental status and amnesia (1.0%)	2.2%
	M3: Bone disorder (4.1%)	10%	Abnormal breathing (5.7%)	12%
	M3: Dementia (3.0%)	6.5%	Vertigo (4.0%)	7.8%
	M3: Hypertension, uncomplicated (11.2%)	23%	Obs. Stroke (0.8%)	1.5%
12	M3: Chronic renal (3.3%)	12%	Tendency to fall (1.3%)	5.5%
	M3: Dementia (3.0%)	11%	Altered mental status and amnesia (1.0%)	3.7%
	M3: Coagulopathy and other blood disorders (5.3%)	14%	Abnormal heart rhythm (0.6%)	1.6%
	M3: Colorectal cancer (1.3%)	3.5%	Fainting (6.3%)	16%
	M3: Bone disorder (4.1%)	11%	Abnormal biochemical result (0.6%)	1.5%
13	M3: Myocardial infarction (5.7%)	65%	Obs. Myocardial infarction (3.4%)	13%
	M3: Angina (7.8%)	73%	Chest pain (12.3%)	43%
	M3: Cardiac disease (other) (9.6%)	82%	Obs. Other cardiovascular disease (2.3%)	3.9%
	M3: Metabolic disorder (11.7%)	66%	Palpitations (1.6%)	1.8%
	M3: Congestive heart failure (6.0%)	22%	Coughing (0.9%)	1.0%
14	M3: Metastatic cancers (1.0%)	4.6%	Fever (1.5%)	5.0%
	M3: Liver disease (moderate or severe) (2.2%)	7.5%	Obs. Cancer (1.2%)	2.9%
	M3: Alcohol abuse (4.5%)	12%	Edema (0.9%)	2.0%
	M3: Other cancers (1.9%)	4.9%	Coughing (0.9%)	1.8%
	M3: Colorectal cancer (1.3%)	3.2%	Nausea and vomiting (1.5%)	2.8%
15	M3: Cardiac valve (4.9%)	54%	Symptom from urination and urinary tract (3.3%)	7.5%
	M3: Congestive heart failure (6.0%)	37%	Tendency to fall (1.3%)	2.6%
	M3: Aortic and other aneurysms (1.9%)	10%	Abnormal breathing (5.7%)	9.5%
	M3: Chronic renal (3.3%)	18%	Obs. Other cardiovascular disease (2.3%)	3.8%
	M3: Cardiac disease (other) (9.6%)	52%	Vertigo (4.0%)	6.2%
16	M3: Chronic pulmonary (12.8%)	70%	Abnormal breathing (5.7%)	22%
	M3: Cardiac arrhythmia (15.4%)	56%	Abnormal heart rhythm (0.6%)	1.2%
	M3: Breast cancer (1.7%)	5.5%	Altered mental status and amnesia (1.0%)	2.0%
	M3: Connective tissue disease (3.6%)	11%	Tendency to fall (1.3%)	2.3%
	M3: Pulmonary circulation disorder (2.4%)	7.3%	Obs. Arrhythmia (0.6%)	1.1%
17	M3: Angina (7.8%)	74%	Abnormal biochemical result (0.6%)	1.8%
	M3: Diabetes (complicated) (6.8%)	62%	Obs. Stroke (0.8%)	2.1%
	M3: Cardiac disease (other) (9.6%)	82%	Obs. Myocardial infarction (3.4%)	8.4%
	M3: Metabolic disorder (11.7%)	78%	Symptom from urination and urinary tract (3.3%)	5.2%
	M3: Myocardial infarction (5.7%)	35%	Tendency to fall (1.3%)	2.0%
18	M3: Diabetes (complicated) (6.8%)	60%	Abnormal biochemical result (0.6%)	2.3%
	M3: Chronic renal (3.3%)	22%	Abnormal breathing (5.7%)	16%
	M3: Metabolic disorder (11.7%)	60%	Altered mental status and amnesia (1.0%)	2.0%
	M3: Muscular peripheral nerve disorders (2.0%)	9.0%	Malaise or fatigue (3.2%)	6.2%
	M3: Other cancers (1.9%)	7.9%	Tendency to fall (1.3%)	2.4%
19	M3: Liver disease (moderate or severe) (2.2%)	16%	Other nonspecific diagnoses (3.7%)	19%
	M3: Metastatic cancers (1.0%)	4.9%	Tendency to fall (1.3%)	4.8%
	M3: Cardiac arrhythmia (15.4%)	66%	Obs. Cancer (1.2%)	4.1%
	M3: Congestive heart failure (6.0%)	25%	Dysphagia and eating difficulties (0.5%)	1.5%
	M3: Coagulopathy and other blood disorders (5.3%)	21%	Abnormal biochemical result (0.6%)	1.7%
20	M3: Congestive heart failure (6.0%)	73%	Obs. Myocardial infarction (3.4%)	7.0%
	M3: Cardiac disease (other) (9.6%)	84%	Tendency to fall (1.3%)	2.7%
	M3: Diabetes (complicated) (6.8%)	58%	Malaise or fatigue (3.2%)	6.0%
	M3: Chronic renal (3.3%)	26%	Abnormal breathing (5.7%)	10%
	M3: Angina (7.8%)	62%	Nausea and vomiting (1.5%)	2.7%

Characteristics with < 1% prevalence in cluster ignores

Abbreviations: M3 = Measuring MultiMorbidity Index

#### Clusters 1–5: No or limited biochemistry

Clusters 1–4 shared a highly increased prevalence of missing biochemical results, meaning that blood tests were rarely or never used. Further, they shared many primary discharge diagnoses: observation for concussion, epistaxis, and dysphagia, among others (Table 3)—conditions that often can be clinically assessed or resolved without the need for biochemical results. Besides that, they differed in age, comorbidities, socioeconomic, and administrative characteristics. Cluster 5 had a high prevalence of missing specific biochemical results, meaning that patients in this cluster often have a small subset of blood tests analyzed, representing a mix of primary discharge diagnosis groups.

#### Clusters 6–9: Younger patients

Clusters 6–9 shared a low average age (mean 32.8–51.4 years) and a need for biochemical tests. Cluster 9 was the smallest (*n* = 2,346; 4.7%) and was notably different with a high mean comorbidity score of 1.25 compared to 0.22–0.27 in Cluster 6–8. These comorbidities were mainly major psychiatric disorders (27%), anxiety and behavioral disorders (23%), alcohol abuse (23%), drug abuse (17%) and epilepsy (14%). Cluster 8 differentiated from 6 to 7 by a high frequency of chest pain as discharge diagnosis (41%). Cluster 6 and 7 had similar increased prevalence of abdominal pain (38% and 33%) and headache (5.6% and 5.2%) but were different in age (mean 32.8 and 50.9) and age-related sociodemographics (cohabitation, educational level, and civil status).

#### Clusters 10–20: Older patients

Clusters 10–20 all had a high average age (mean 68.8–83.3 years). Clusters 10 and 11 described older patients with low comorbidity, notably a low prevalence of cardiovascular, metabolic, and pulmonary comorbidities. Cluster 10 was mainly males discharged with observation for cancer, symptoms from urination and urinary tract, and fever. Cluster 11 was primarily females presenting with a tendency to fall, altered mental status, and abnormal breathing. Cluster 12 had an increased prevalence of tendency to fall, altered mental status and amnesia, and fainting. Further, Cluster 12 had the highest mean age but only a moderate comorbidity burden. Cluster 13 patients were mainly discharged with nonspecific chest pain and observation for myocardial infarction, had a high prevalence of cardiovascular comorbidities, and low occurrence of elevated plasma troponin and d-dimer. Cluster 14 had a mix of discharge diagnoses but differed by comorbidity profile with an increased prevalence of alcohol abuse, liver disease, and metastatic cancers.

Clusters 15–20 were all small in size (*n* = 414–1324) and had high levels of comorbidity. They had increased prevalence of tendency to fall and abnormal breathing as discharge diagnoses across all clusters, but also increased prevalence of observation for stroke, altered mental status, observation for myocardial infarction, abnormal heart rhythm, nausea and vomiting, malaise and fatigue, abnormal biochemical results, and symptoms from urinating and urinary tract across most clusters in different combinations. Likewise, these clusters had a high level of comorbidity with increased prevalence of different cardiovascular, metabolic, pulmonary, hematologic, renal, hepatic, malignant, and substance abuse in different combinations across the clusters.

### Prognosis

The overall risk of 30-day readmission and mortality for the entire cohort was 10.1% (9.8–10.4%) and 1.3% (1.2–1.4%), respectively, but differed notably across clusters (Table 2). The risk of readmission ranged from 4.4% (3.9–4.8%) to 27% (24–30%) across the clusters and 3.2% (2.1–4.9%) to 23% (20–27%) across the 32 different nonspecific discharge diagnosis groups (Table 4). When combining grouping of the discharge diagnosis and the assigned cluster, it was possible to further differentiate the risk of readmission for the patients (Table 4). For instance, the overall risk of readmission for patients discharged with nonspecific abdominal pain was 16% but differed across cluster assignment from 3% in Cluster 6 to 26% in Cluster 12. Similarly, the overall risk of readmission for Cluster 11 was 11% but differed from 3% if discharged with fainting to 15% if discharged with abnormal breathing, abdominal pain, or tendency to fall. The outcome estimates were similar across the train and test data sets with small variations (Supplementary Table S5).

**Table 4:** Differentiated risk of readmission by combining cluster and primary nonspecific discharge diagnosis

Nonspecific discharge diagnosis group	*n*	Total cohort Pct. (95% CI)	1	2	3	4	5	6	7	8	9	10	11	12	13	14	15	16	17	18	19	20
Total cohort	50,000	10% (10–10%)	4.5%	5.4%	12%	18%	8.8%	6.4%	5.9%	4.4%	15%	15%	11%	19%	11%	26%	18%	17%	15%	16%	27%	24%
Headache, unspecified	1642	7.6% (6.4–8.9%)	6.3%	6.9%			5.7%	7.8%	5.9%	*			11%									
Obs. Stroke	422	8.7% (6.3–12%)																				
Fainting	3132	6.8% (6.0-7.7%)	*	*			4.5%	2.9%	3.5%	2.8%		7.7%	3.4%	12%								
Obs. Nervous system disorder, unspecified	605	7.2% (5.7–9.2%)							*													
Seizures or muscle cramps	827	11% (9.0–13%)						6.3%			19%											
Symptoms involving the nervous and musculoskeletal systems	1468	7.8% (6.5–9.4%)					5.0%	6.0%	4.3%			11%	14%									
Vertigo	2019	7.6% (6.5–8.8%)		5.9%			8.2%	*	4.8%	6.4%		8.1%	8.1%									
Obs. Concussion	909	8.3% (6.6–10%)	2.5%	*	11%	15%																
Epistaxis and oropharyngeal bleeding	854	13% (11–15%)		6.8%	8.6%	13%																
Chest pain	6156	5.2% (4.7–5.8%)	2.0%	2.1%	7.2%		6.7%	2.3%	2.6%	3.0%	8.4%	8.9%	5.9%		10%		19%	10%				
Observation for suspected myocardial infarction	1690	6.0% (4.9–7.3%)					5.6%		*	1.5%			7.0%		9.4%							
Palpitations	811	3.2% (2.1–4.9%)						*	*	*												
Abnormal heart rhythm	276	10% (7.3–14%)																				
Obs. Arrhythmia	298	7.4% (4.9–11%)																				
Obs. Other cardiovascular disease	1130	7.0% (5.6–8.6%)							3.8%	3.9%			6.0%									
Coughing	450	10% (7.3–12%)																				
Abnormal breathing	2836	16% (15–17%)				26%		3.1%	6.0%	5.9%	16%	21%	15%	29%				23%		18%		
Abdominal pain	8559	11% (10–12%)	7.7%	7.8%	16%			8.2%	7.8%	8.9%	16%	19%	15%	26%		27%		19%	16%			
Nausea and vomiting	726	18% (15–21%)																				
Dysphagia and eating difficulties	258	16% (12–20%)																				
Altered mental status and amnesia	500	19% (16–23%)											14%									
Edema	450	11% (8.4–13%)																				
Fever	750	17% (15–19%)							11%			11%										
Malaise or fatigue	1590	11% (9.5–12%)						5.3%	5.0%	4.0%			11%									
Tendency to fall	650	16% (13–19%)											15%									
Abnormal findings of the skin	441	6.3% (4.5–8.9%)																				
Symptom from urination and urinary tract	1645	19% (17–21%)		11%	14%	22%						21%										
Acute pain	2232	8.6% (7.5–10%)	4.5%	6.1%	10%	12%		5.2%	3.9%	5.8%			8.0%									
Obs. Suspected disease or condition	3932	10% (8.9–11%)	1.8%	4.9%	12%	19%	10%	6.6%	5.6%	6.3%	17%	15%	10%	17%								
Other nonspecific diagnoses	1844	15% (13–17%)	4.8%	*				7.1%	11%	4.9%		18%	12%								32%	
Abnormal biochemical result	312	16% (13–20%)																				
Obs. Cancer	586	23% (20–27%)										21%										

Cells with < 100 observations blanked, cells with < 5 events censored (*)

Abbreviations: CI = confidence interval

## Discussion

A large proportion of patients is discharged without disease-specific diagnoses after acute hospital care. Nonspecific discharge diagnoses constitute about 50% of discharges after shorter stays and a notable proportion of adverse outcomes, which indicate missed diagnoses [2]. Thereby, patients discharged from acute hospital care with nonspecific diagnoses represent a relevant point of improvement. However, research within this population has been scarce, possibly due to significant heterogeneity. With this study, we have utilized unsupervised machine learning models on multi-dimensional, inhomogeneous, register-based data of acute patients discharged with nonspecific discharge diagnoses and identified 20 clusters. The clusters expressed different clinically recognizable groups describing different nonspecific diagnoses, patient characteristics, and prognoses.

This clustering approach can aid in better differentiation and understanding of patients with nonspecific discharge diagnoses. Identification of patients with a poor prognosis or need for further diagnostics can be difficult in a heterogeneous population with an overall low risk of readmission, warranting further differentiation. A binational study of COVID-19 patients at different stages of disease progression showed improved Area Under the Receiver Operating Curve for sensitivity and positive predictive value when predicting need for ICU stay for admitted patients compared to all COVID-19 positive patients [36]. The same was observed, when predicting ventilator treatment and death for patients just before ICU transfer compared to at admission or at test positivity. Thereby, predictive performance can be improved by predicting in more homogenous populations. Further, the most important predictors also changed from more general variables such as age, BMI, and hypertension at test positivity to temporal variables, such as blood gas results, vital signs, or biochemical variables [36]. Another study has investigated 30-day emergency admission from the NHS Secondary Uses Service data and revealed slightly better sensitivity and positive predictive values by conducting unsupervised clustering prior to supervised modeling, rather predictions in groups stratified by comorbidity only [22]. Based on these studies, it is likely that our clustering approach can assist in forming delimited subgroups yielding better predictions and identification of more personalized risk factors. These risk factors or predictions in combination with homogenous patient clusters could also allow for interventions and prevention strategies to reduce missed diagnoses and patient harm. Nevertheless, as the model was developed solely on administratively collected health-care data, which is not always readily available in a clinical context, this model is not currently fit for clinical testing or implementation. Despite this limitation, the methodological approach show promise, that needs further testing in other settings and with other data sources.

The 20 identified clusters followed some overall structure, with Clusters 1–4 sharing no biochemistry and related diagnoses, Cluster 5 of patients having a selected subset of biochemistry, Clusters 6–9 describing younger patients, and Clusters 10–20 describing older patients. Cluster 1–4, 6–8, 11, 13, 14, and 18 all shared a predominance of LOS < 24 h, identifying them as mainly “ED clusters”, whereas the remainder mainly constituted acute in-hospital care after ED transfer. As EDs are still transitioning with the 2018 implementation of Emergency Medicine in Denmark, they still operate very differently causing the need for including longer hospital stays as well. Within the overall structure, the cluster differed in terms of different comorbidities, nonspecific discharge diagnoses, and more. For instance, Cluster 9, despite being a cluster of mainly younger patients, showed a high mean comorbidity score with a high degree of substance abuse, psychiatric and neurological comorbidities—a different pattern than the other clusters. Further, the risk of mortality was also higher than in the other clusters of young patients, and the risk of readmission aligned with clusters of older patients. A higher mean comorbidity burden did not always result in a worse prognosis, as Cluster 13 (patient discharged with nonspecific chest pain with known cardiovascular comorbidity) had a moderate comorbidity score but a low risk of mortality and average risk of readmission. This highlights that age and comorbidity score by themselves are not always sufficient for risk stratification and that discharge diagnoses and pattern of specific comorbidities aided in forming different clusters with differences in prognoses. However, the clusters of patients with both low age and low comorbidity score (Clusters 1, 2, 6, 7, and 8; comprising 51% of the cohort) all had a low risk of adverse outcomes. In a clinical setting, patients with nonspecific diagnoses from these clusters should be of focus for brief evaluations and discharges to avoid ED overcrowding or unneeded examinations. Patients from the clusters with above-average risks might benefit from further examinations. A cause for the risk of adverse outcomes might be diagnostic errors where diagnoses are missed, wrong or delayed [10]. Harms from diagnostic errors at discharge could potentially be reduced with individualized initiatives such as prolonged stay or planned follow-up in the days following discharge.

Some clusters expressed an apparent relation between the nonspecific diagnosis and comorbidities. In Cluster 13, there was a large proportion of patients with previous ischemic heart conditions that were discharged with nonspecific chest pain or observation for myocardial infarction, clinically supported by normal plasma troponins. Cluster 16 described a phenotype of patients with chronic obstructive pulmonary disease (COPD) often discharged with nonspecific breathing problems, which were not outright COPD exacerbation, pneumonia, or other specific disease, but rather represent mild symptoms or anxiety. In agreement with previous research, this cluster also expressed an increased prevalence of osteoporosis [37]. The model also revealed clusters on the same disease trajectory but at different stages of disease. Cluster 13, 17, and 20 had notable cardiovascular disease and co-occurring heart failure, diabetes, chronic renal disease, and hematological diseases, associations previously established [38]. Similarly, Cluster 9, 14, and 19 all shared a substantially increased prevalence of alcohol abuse and liver disease, where the youngest (Cluster 9) had an increased prevalence of psychiatric comorbidities and drug abuse as opposed to an increased frequency of cancers, especially metastatic, and diabetes in the older. Excessive alcohol consumption is known to cause alcohol-related liver disease and has established social inequality for most age groups [39]. Even though discharge diagnoses were part of the clustering model, the different nonspecific discharge groups were observed in many different clusters. Further, we still observed variations in the risk of readmission across discharge diagnoses inside a cluster and for discharge diagnoses across clusters. This supports, that discharge diagnosis by itself is not sufficient to differentiate the patients and that the risk of adverse outcomes is affected by the patient’s other characteristics.

The identified clusters follow a pattern corresponding to other studies. Latent class clustering of acutely hospitalized medical patients over 65 years old showed 10 clusters of patients (6 shared between the sexes and two unique for males and females) [17]. Women had a “Degenerative and mental disorders” and “Complex multimorbidity” comorbidity pattern not detected in men, comparable to our Clusters 12 and 20, respectively. For men, unique patterns of “Mental disorders” and “Dementia and Parkinson’s Disease” were discovered. In our analysis, “Dementia and Parkinson’s Disease” might correspond to Cluster 18 and 19, predominantly including males with an increased prevalence of dementia and other neurodegenerative diseases, but also other comorbidities. In our analysis, mental disorders were mainly found in Cluster 9 of younger patients with a male overweight and co-occurring alcohol and drug abuse. Healthcare complexity and utilization prior to admission and risk of readmission and mortality after discharge differed between the clusters. Similarly, an Australian study clustering the entire ED population based on a total of 16 sociodemographic, clinical, and time-related variables identified 6 clusters [11]. We believe that a larger variation of characteristics causes the higher number of clusters in the present study. In concordance with other studies, we also discovered clusters of otherwise healthy patients [40, 41]. 

In future studies, distinguish between symptom-diagnoses (e.g., chest pain), observation diagnoses (e.g., observation for concussion) and nonspecific problem diagnoses (e.g., epistaxis or urinary retention) could be beneficial, as they represent different degrees of diagnostic uncertainty. Automated systems for identifying symptom-based diagnoses have previously been tested [42]. However, we have previously demonstrated that R- and Z03-diagnoses have similar characteristics and outcomes [2]. Some diagnosis groups, like chest pain and observation for myocardial infarction, may indicate the same underlying symptom. Validation studies assessing if nonspecific diagnoses actually represent diagnostic uncertainty at discharge also needs to be conducted. Further work with the identified clusters is also warranted, for instance identification of differences in proteomics, genetics or other biomarkers supporting an inherent difference between the clusters.

### Strengths and limitations

The strengths of this study include a large cohort of patients with many available variables relevant for clustering. The Danish registers include full, prospectively collected data for all individuals, ensuring complete follow-up, and are recognized for high validity and completeness [24, 26]. We tested two different clustering methods and used evaluation by experts of different backgrounds to determine the best model and to ensure clinical relevance. We detected a poor silhouette score, which might be due to too high dimensionality—dimensionality reduction techniques or feature selection might have improved the performance [43]. We sought to limit the “curse of dimensionality” by including selected variables from different categories with limited expected covariance [43]. However, some variables express the same information for a subset of the cohort, e.g., whether blood samples were analyzed or not, thereby overvaluing these variables as an isolated cluster.

Other limitations include a lack of more clinical variables. Abnormal and incomplete vital signs at discharge with nonspecific diagnoses after ambulance transport have been found to be associated with an increased risk of 30-day mortality with a corresponding adjusted odds ratio of 6 for non-critical and an odds ratio of 48 for critical deviation [44, 45]. Therefore, it would be relevant to include in clustering models or inclusion criteria, along with triage, primary complaints, and findings during diagnostic work-up. Further, we were limited by some methodological choices. While also interesting in an ED perspective, we needed to excluded stays < 3 h to ensure that out-of-hours contacts were not included. We sought to mimic a “normal ED population” by excluding patients where the diagnostic course was markedly different, hindered, limited or rather represented psychiatric conditions. However, this might limit the external validity and must be considered when comparing the results to other populations. We defined readmissions as lasting at least 12 h to exclude brief re-evaluations or planned outpatient visits and to focus substantial problems, needing prolonged observation or treatment. Thereby, we sought to focus on readmissions likely caused by missed diagnoses potentially leading to patient harm. However, minor missed diagnoses might have been omitted by this criterion, possibly causing under-estimation of the outcome. To ensure that we only included patients that were discharged alive, we excluded patients dying the day of discharge, which also possibly and unintentionally excluded patients dying at home. It is possible that some patients where treatment is deemed futile are discharged to die at home without full diagnostic work-up, resulting in a nonspecific discharge diagnosis [45]. We sought to minimize the effect of these expected deaths by excluding patients in registered palliative care since the median expected lifetime of patients terminally ill or receiving palliative care is under two months [46, 47]. In a clinical setting, nonspecific diagnoses should be used for patients discharged without an established diagnosis, but the validity has yet to be determined.

## Conclusion

We have employed two unsupervised machine learning techniques on patients discharged with nonspecific diagnoses, a heterogeneous population of patients discharged without an established diagnosis. A k-prototypes model identifying 20 clusters best fitted our data, revealing recognizable subgroups of patients with differentiated characteristics and prognosis. The different nonspecific discharge diagnosis groups were distributed across multiple clusters, showing the importance of not only considering the diagnosis but also other patient characteristics. This clustering approach has shown potential for differentiation of the patients, enabling further research in delimited populations, with the possibility of developing better prediction models in cluster-then-predict approaches.

## Supplementary Information

Below is the link to the electronic supplementary material.

Supplementary Material 1

## Data Availability

Due to Danish legislation, the datasets supporting the current findings cannot be shared or made publicly available. The same data can be accessed by researcher at authorized Danish institutions. The used codes used in SAS and python can be shared upon reasonable request.

## Declarations

### Presentations

Initial results from this paper has been presented as an ePoster at Bispebjerg and Frederiksberg Hospital annual research day 2023 (Lassendagen).

## Abbreviations

DNPR: Danish National Patient Register
ED: Emergency department
ICD-10: International Classification of Diseases, 10th revision
ICU: Intensive care unit
M3: Multimorbidity Measure
