# Supplementary material for: Identifying 20 homogeneous clusters of acute patients discharged with nonspecific diagnoses through k-prototypes mixed data clustering
Source: BMC Emerg Med. 2026 Jan 10;26:44. doi: 10.1186/s12873-025-01459-7 (PMC12882614; doi:10.1186/s12873-025-01459-7)
Supplement: Supplementary file 1 — Supplementary Material 1 [file 12873_2025_1459_MOESM1_ESM.docx]

Supplementary material

**Identifying 20 homogeneous clusters of acute patients discharged with nonspecific diagnoses through k-prototypes mixed data clustering**

Rasmus Gregersen Mottlau^1,2,3^; Marie Villumsen^2^; Axel Nyström^4^; Hanne Nygaard^1,3^; Jens Rasmussen^1^; Mikkel B. Christensen^5,6,7^; Jakob Lundager Forberg^8^; Janne Petersen^2,3^

**Affiliation**

^1^ Department of Emergency Medicine, Copenhagen University Hospital – Bispebjerg and Frederiksberg, Copenhagen, Denmark

^2^ Center for Clinical Research and Prevention, Copenhagen University Hospital – Bispebjerg and Frederiksberg, Copenhagen, Denmark

^3^ Department of Public Health, Faculty of Health and Medical Sciences, University of Copenhagen, Copenhagen, Denmark

^4^ Department of Laboratory Medicine, Lund University, Lund, Sweden

^5^ Copenhagen Center for Translational Research, Copenhagen University Hospital – Bispebjerg and Frederiksberg, Copenhagen, Denmark

^6^ Department of Clinical Pharmacology, Copenhagen University Hospital – Bispebjerg and Frederiksberg, Copenhagen, Denmark
^7^ Department of Clinical Medicine, University of Copenhagen, Copenhagen, Denmark

^8^ Department of Emergency Medicine, Helsingborg Hospital, Helsingborg, Sweden

**Corresponding author:**

Rasmus Gregersen Mottlau, MD.
Department of Emergency Medicine, Copenhagen University Hospital – Bispebjerg and Frederiksberg, 2400 Copenhagen NV.
Email: rasmus.gregersen.mottlau@regionh.dk

# Supportive methods

## Model details

For clustering, we employed both partitional and hierarchical algorithms. The k-prototypes algorithm, a modified k-means algorithm combining categorical and continuous variables, was chosen as the partitional model.^1^ We tested 1 to 30 number of clusters and for each number of cluster, we initiated 30 different random initiations with a maximum of 100 iterations and chose the best initiation based on cost function optimization, evaluating Euclidean dissimilarity for continuous variables and matching dissimilarity for categorical variables.^1^ Initializations were done using the methods presented by Cao et al, suggesting initial centroids based on categorical density rather than frequency.^2^ To calculate the Silhouette score, we computed a mixed distance matrix.^3^ For this, we initially computed a Euclidian distance matrix for numerical variables and a matching distance matrix for categorical variables and calculated the average distance sums as 8.0 and 16.5, respectively. A mixed distance matrix was computed by adding the two distance matrixes. Based on the overweight of categorical features in the dataset, we wanted to maintain a categorical overweight in the combined distance matrix. From this reasoning, we tested overweighting the categorical distance matrix by none, 2 or 3, resulting in continuous:categorical distance ratios of 1:2.1, 1:4.1, and 1:6.2, respectively. The Silhouette score describes similarity within a cluster compared to dissimilarity to other clusters. Likewise, the k-prototypes algorithm weights the categorical dissimilarities relative to numerical dissimilarities by a gamma-coefficient. We tested gamma-coefficients of 1, 2, and 3. For hierarchical clustering, we employed agglomerative clustering, where each observation is initially considered its own cluster.^4^ The process then stepwise combines the clusters with the least difference (distance) until all observations are gathered in a single cluster—also known as a ‘bottom-up’ approach. The differences across clusters and observations were based on the same mixed distance matrix as used for k-prototypes. We employed the Ward linkage methods and Euclidean distance measures.^5^ We identified final model candidates based on scree plots (plotting the total sum of squared distance within clusters by number of clusters) for the partitional clustering and dendrograms (plotting distances before clusters are merged) for the hierarchical clustering, supplemented by Silhouette score plots.

# Supportive figures

## Figure S1

Scree plots of train data (n=50,000) and test data (n=42,650) of k-prototypes cluster 1 to 24 for gamma-coefficient values 1, 2 and 3.


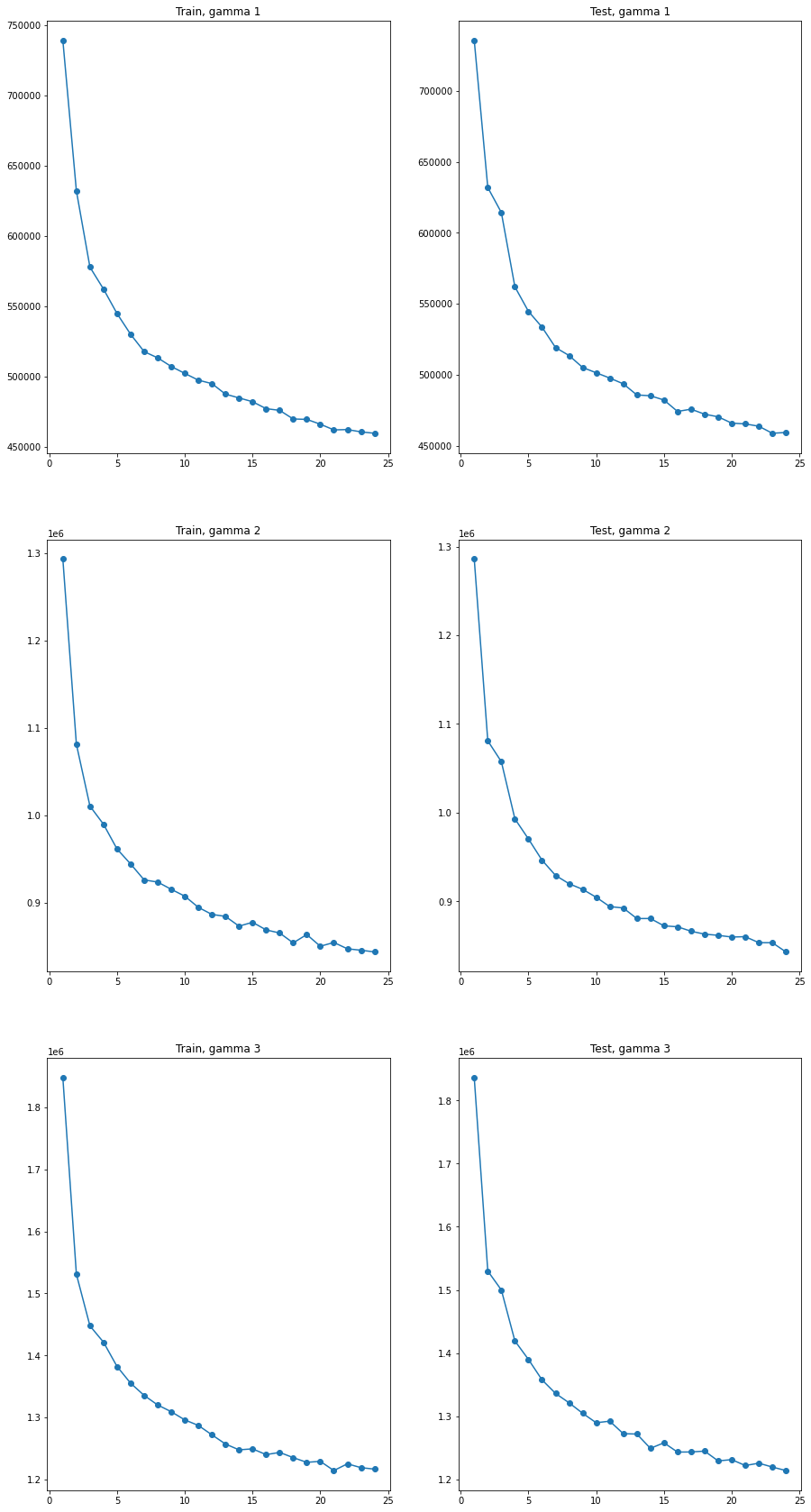


## Figure S2

Silhouette score plots of train data (n=50,000) and test data (n=42,650) of k-prototypes cluster 1 to 24 for gamma-coefficient values 1, 2, and 3. Mixed distance matrix formed with a categorical weight of 2 for all models.


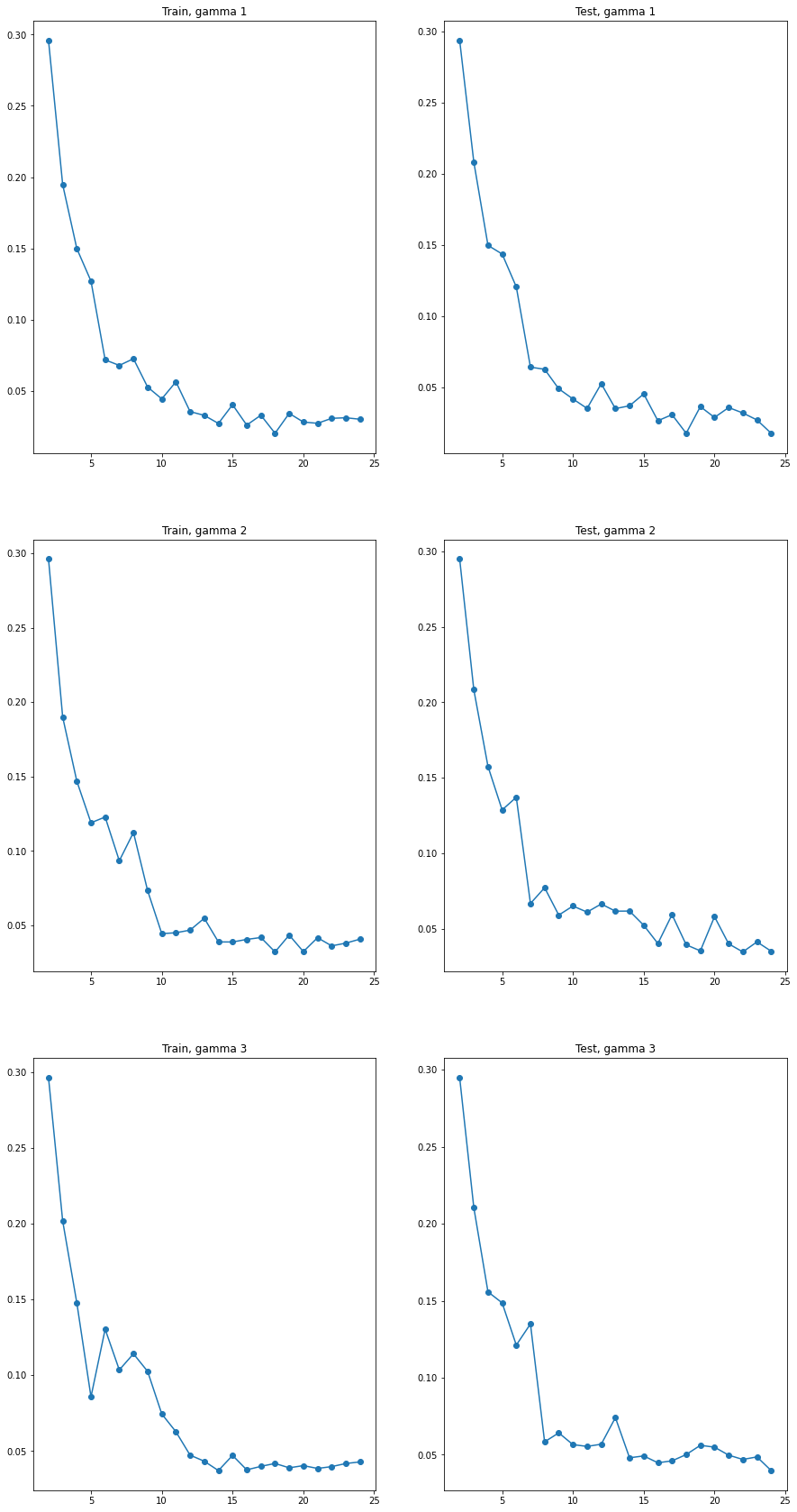


## Figure S3

Dendrogram of train data (n=50,000) using agglomerative clustering on a mixed distance matrix with categorical weight 2. Three suggestive cut-offs are marked at 11 (red), 19 (blue) and 22 (green) clusters.


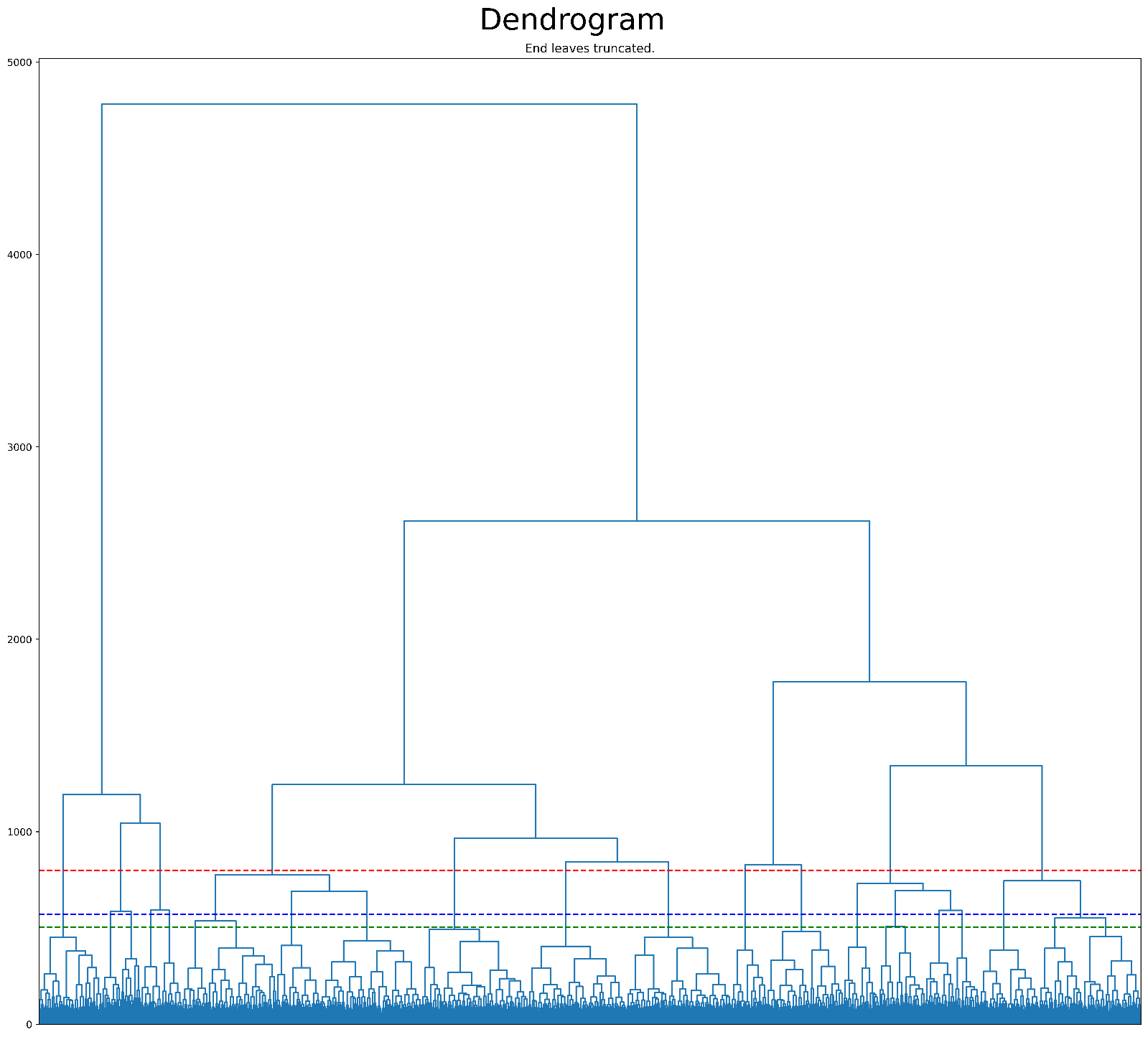


**Figure S4**. Heatmap of M3 comorbidities showing the relative prevalence of a feature (row) in a cluster (column) compared to the entire cohort. The color intensity represents lower (blue), similar (white), and elevated (red) logarithmic relative prevalence.


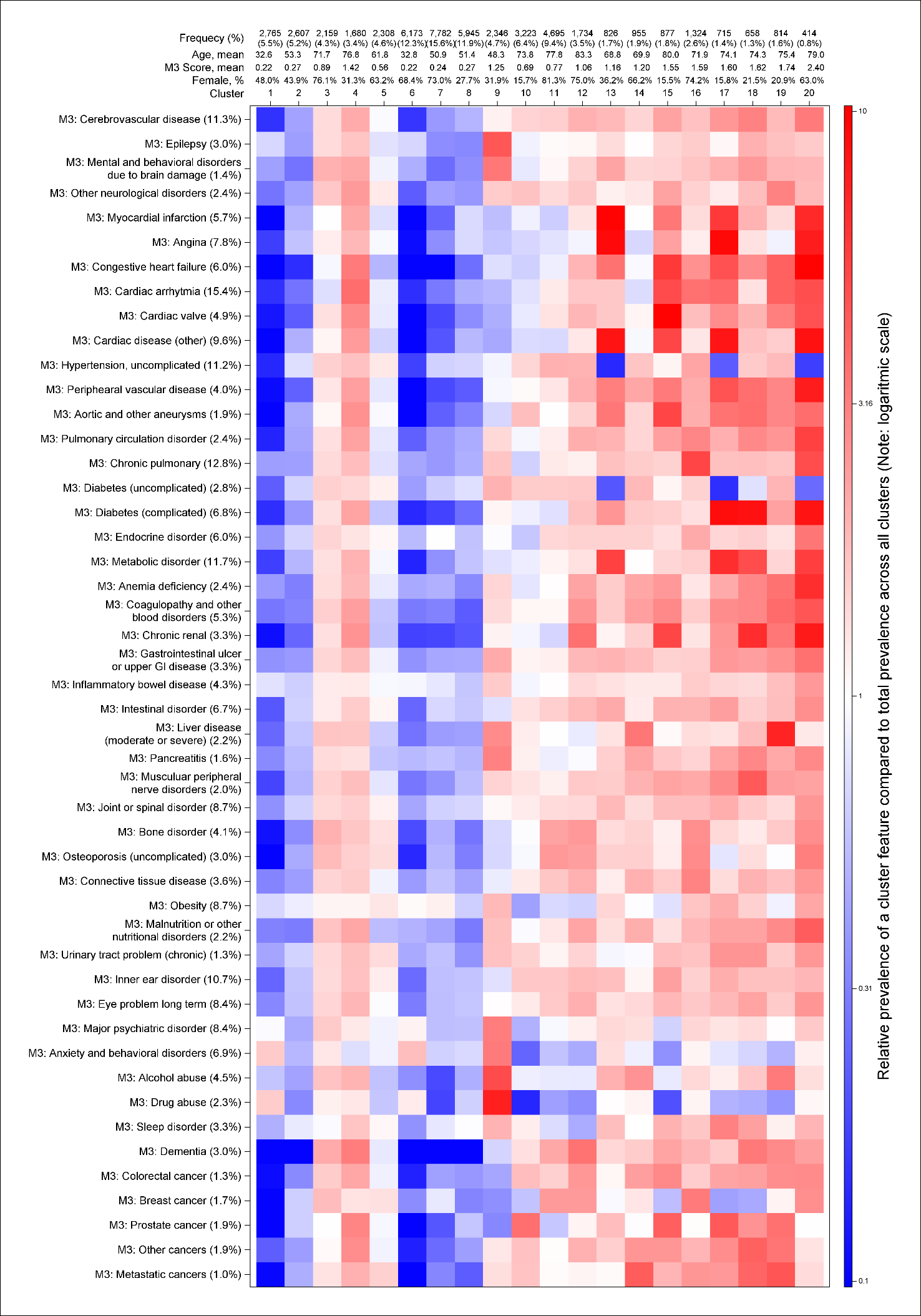


**Figure S5**. Heatmap of nonspecific diagnoses groups showing the relative prevalence of a feature (row) in a cluster (column) compared to the entire cohort. The color intensity represents lower (blue), similar (white), and elevated (red) logarithmic relative prevalence.


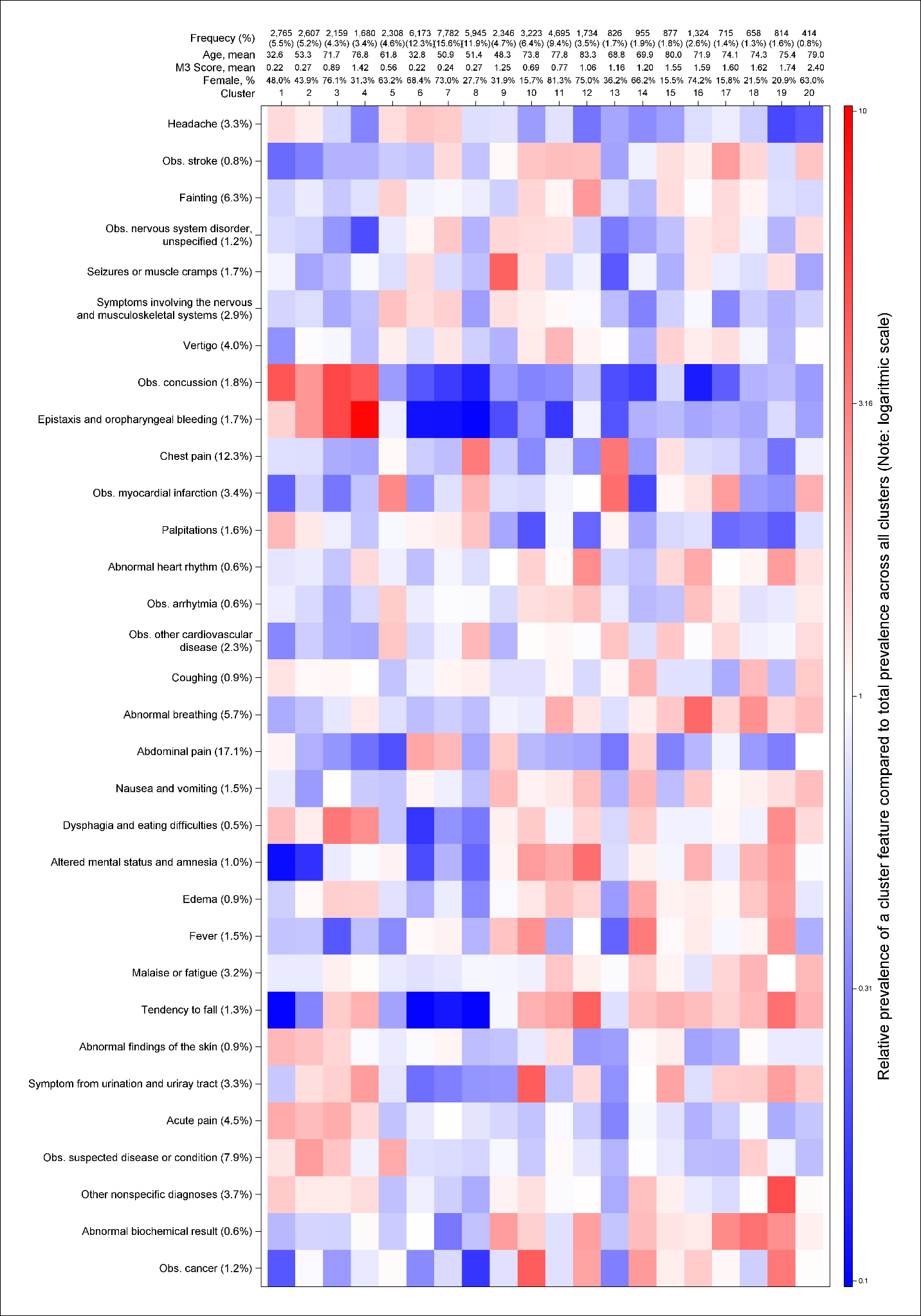


**Figure S6**. Heatmap of sociodemographic and administrative information showing the relative prevalence of a feature (row) in a cluster (column) compared to the entire cohort. The color intensity represents lower (blue), similar (white), and elevated (red) logarithmic relative prevalence.


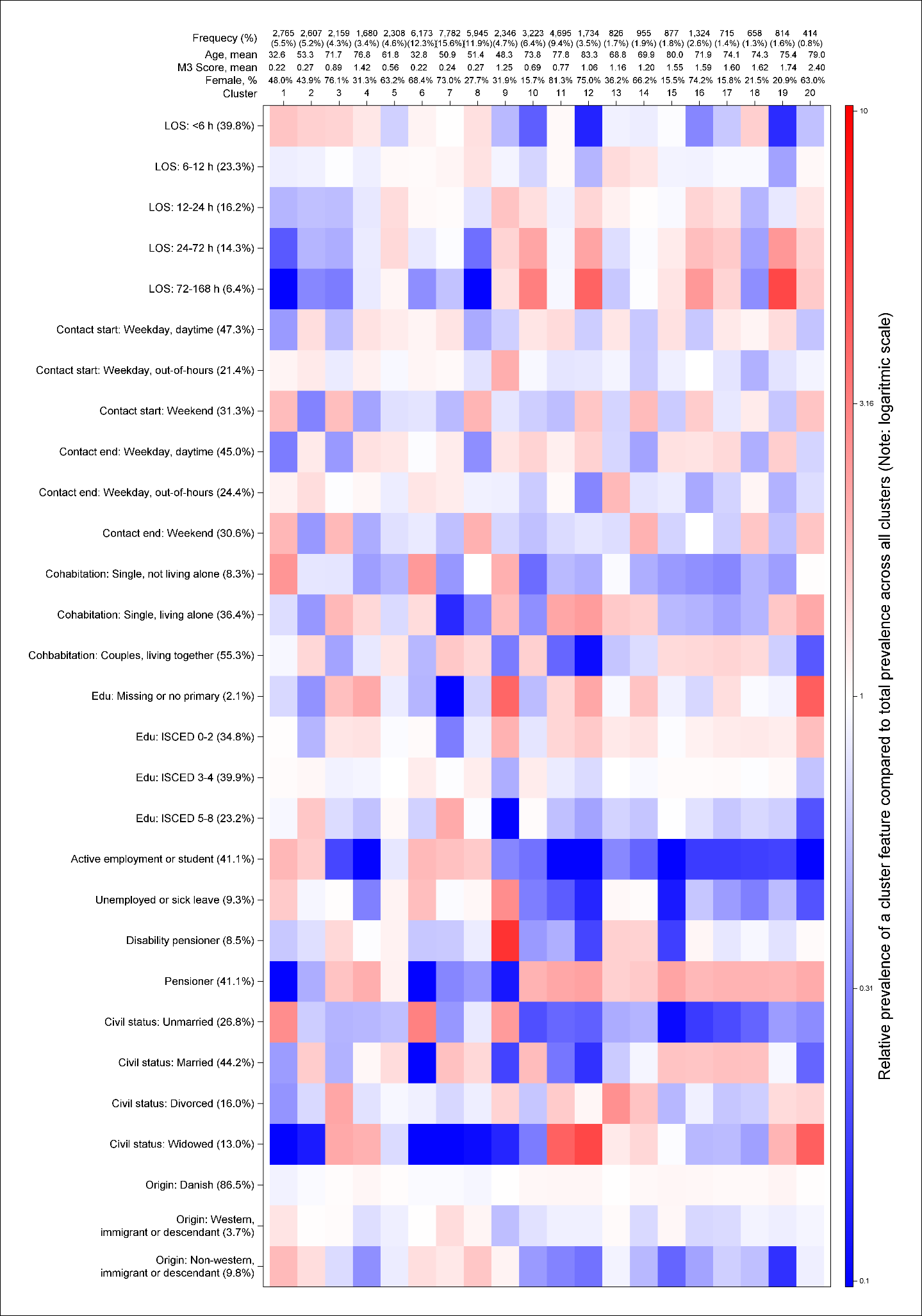


**Figure S7**. Heatmap of laboratory results showing the relative prevalence of a feature (row) in a cluster (column) compared to the entire cohort. Patients without laboratory results omitted. The color intensity represents lower (blue), similar (white), and elevated (red) logarithmic relative prevalence.


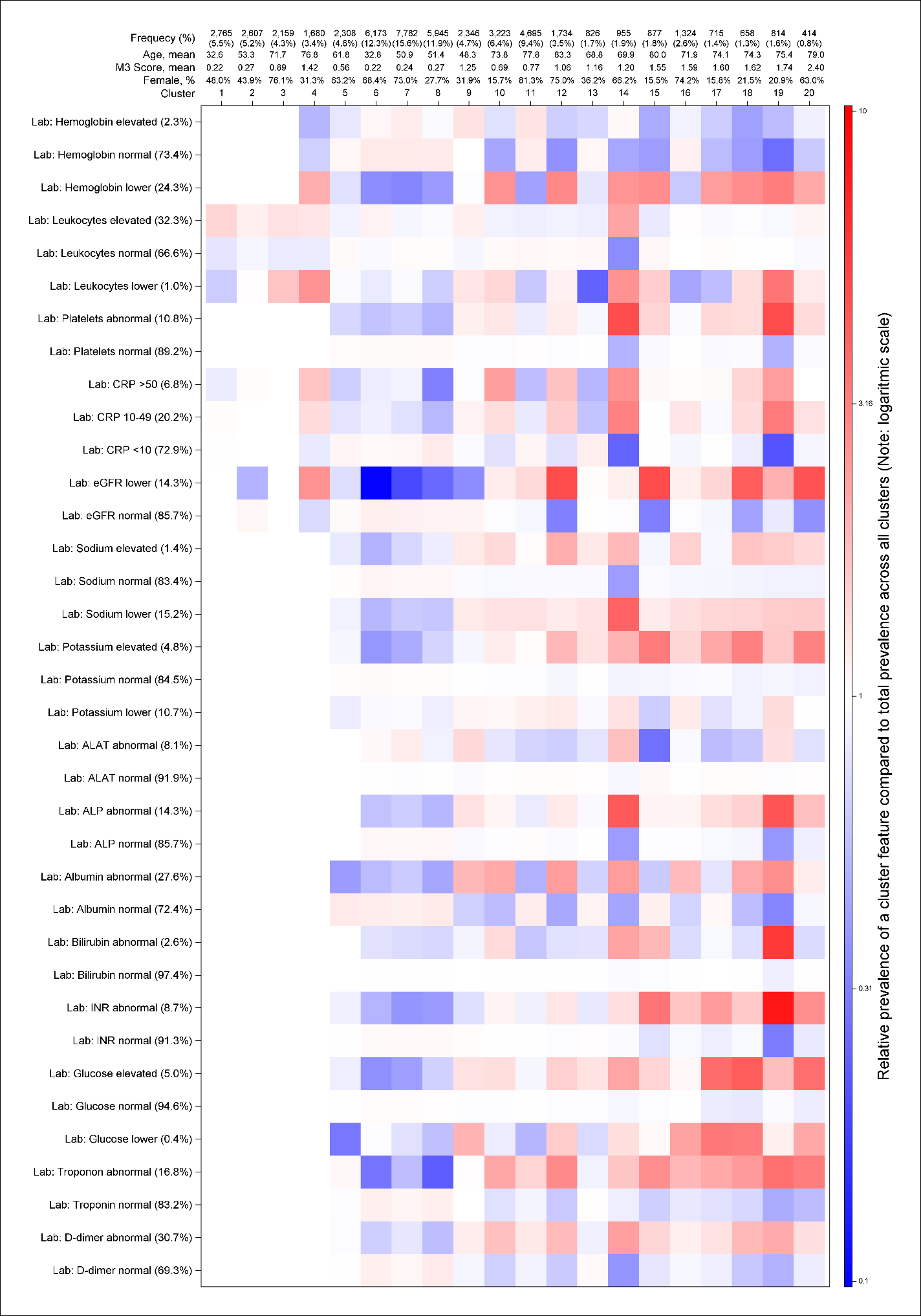


**Figure S8**. Heatmap of missing laboratory results showing the relative prevalence of a feature (row) in a cluster (column) compared to the entire cohort. The color intensity represents lower (blue), similar (white), and elevated (red) logarithmic relative prevalence.


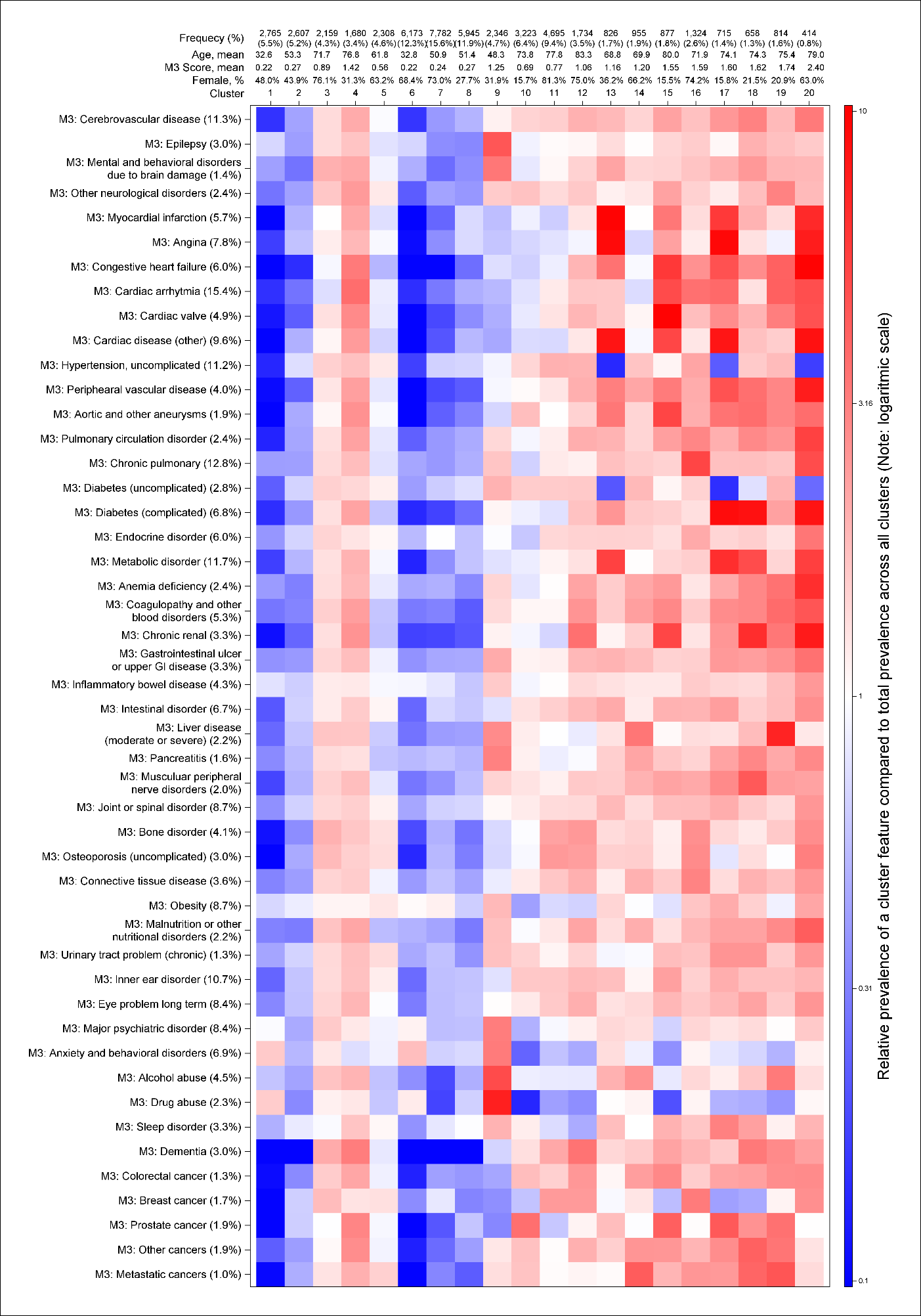


**Figure S9**. Condensed heatmap of the relative prevalence of selected features (rows) in a cluster (columns) compared to the entire cohort prevalence in the test data set. The color intensity represents lower (blue), similar (white) and elevated (red) relative prevalence using a logartimic color scale.


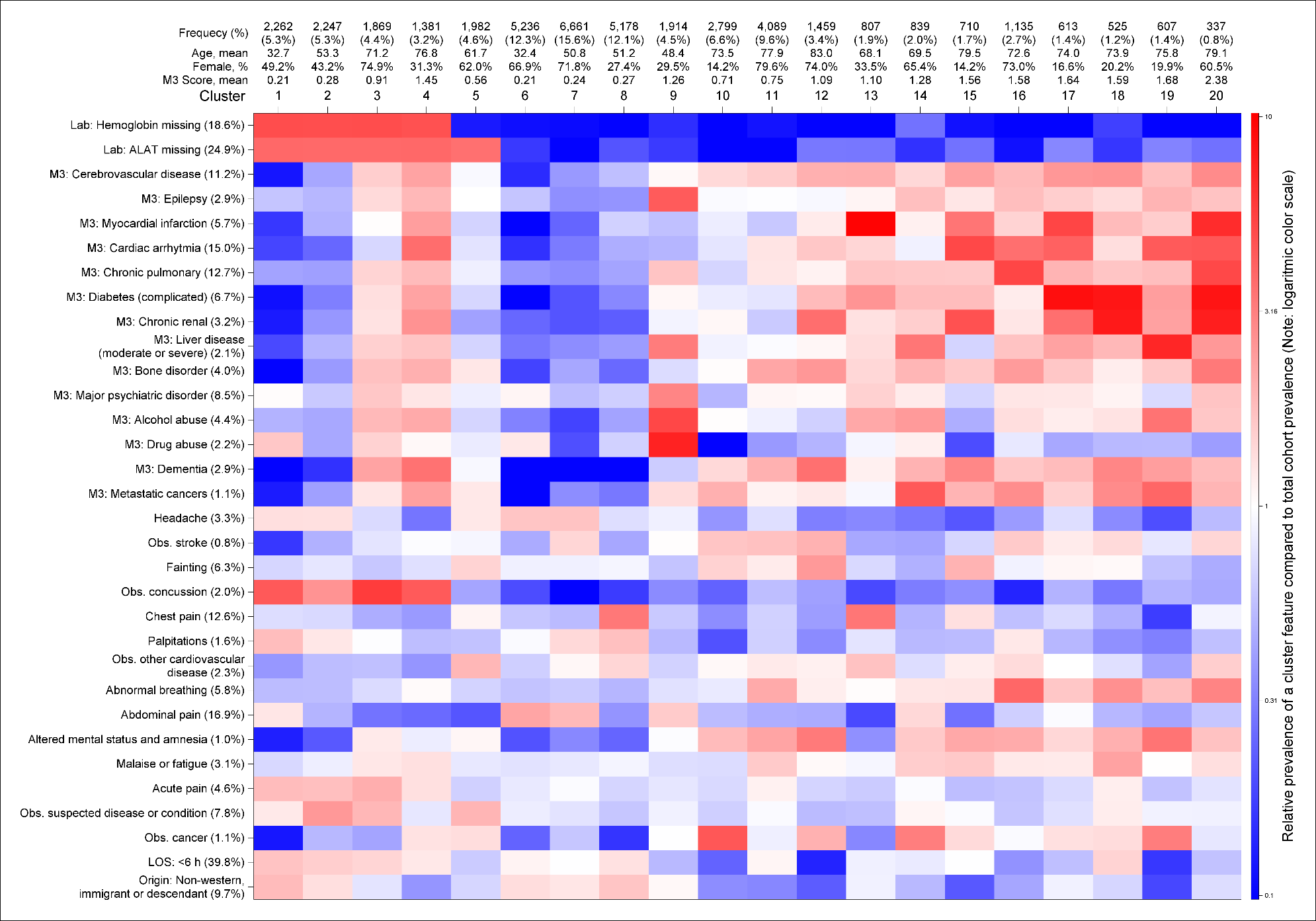


# Supportive tables

## Table S1

Diagnoses from the R and Z03 chapters, that were considered disease-specific or administrative and therefore not included.

| ICD-10 code | Definition |
| --- | --- |
| DR029 | Gangrene, not elsewhere classified |
| DR091 | Pleuritis, not elsewhere classified |
| DR092 | Respiratory failure |
| DR092A | Cardiopulmonary failure, unspecified |
| DR392* | Uremia |
| DR570* | Shock |
| DR568F | Oligoepilepsy |
| DR67* | Barthel Index registrations |

* Including subgroups

## Table S2

Exclusion criteria definitions

| Criteria | Definition |
| --- | --- |
| Left against medical advice | ICD-10 code: DZ766* |
| Diagnoses related to pregnancy, birth or the postpartum period, during the acute contact | ICD-10 codes: DO*, DZ3*, DZ038O, DZ038M |
| Registered palliative care prior to the acute contact | ICD-10 code: DZ515* Procedure-code: BXB* |

* Including subgroups

## Table S3

Characteristics grouping and definitions for sociodemographic and administrative variables.

| Variable | Levels | Definitions |
| --- | --- | --- |
| **Socioeconomic**  Most recent information prior to the acute contact, most often at 1^st^ of January in the same year. Imputed with previous or coming year if missing. | | |
| **Cohabitation** Based on the variable familie_type and hustype from the population statistics register (BEF) | Single, not living alone | Familie_type: 5, 9 or 10 AND hustype: 1, 2 or 5 |
|  | Single, living alone | Familie_type: 5, 9 or 10 AND hustype: 3, 4, 6 |
|  | Couple, living together | Familie_type=: 1, 2, 3, 4, 7, 8 |
| **Educational level**  Based on the variable HFAUDD (highest completed education), grouped by International Standard Classification of Education (ISCED) from the Danish Education Register | Missing or no primary education |  |
|  | Low educational level | ISCED 0-2 |
|  | Medium educational level | ISCED 3-4 |
|  | High educational level | ISCED 5-8 |
| **Socioeconomic classification**  Based on the variable pre_socio (major source of income) from the Income Statistics Register and grouped by the socio13 scheme | Active employment or student | 310, 110-114, 120, 131-135, 139 |
|  | Unemployed or sick leave | 210, 220, 330 |
|  | Disability pensioner | 321 |
|  | Pensioner | 322, 323 |
| **Marital status (civil status)**  Based the variable civst from the population statistics register (BEF) | Unmarried | U |
|  | Married | G, P |
|  | Divorced | F, O |
|  | Widowed | E, L |
| **Origin**  Based on the variable opr_land (country of origin) | Danish | Denmark |
|  | Western, immigrant or descendant | EU-countries, Andorra, Iceland, Liechtenstein, Monaco, Norway, San Marino, Great Britain, the Vatican State, Canada, USA, Australia and New Zealand |
|  | Non-wester, immigrant or descendant | Remaining countries |
| **Administrative**  Information about the acute hospital course | | |
| **Length of stay**  Total length of stay including department transfers | 3-6 hours |  |
|  | 6-12 hours |  |
|  | 12-24 hours |  |
|  | 24-72 hours |  |
|  | 72-168 hours |  |
| **Start of contact**  Categorized end time of the hospital course | Weekday, daytime | Monday–Friday, 8:00 to 18:00 |
|  | Weekday, out-of-hours | 18:00 to 8:00 starting Monday–Thursday |
|  | Weekend | Friday 18:00 to Monday 8:00 |
| **End of contact**  Categorized end time of the hospital course | Weekday, daytime | Monday–Friday, 8:00 to 18:00 |
|  | Weekday, out-of-hours | 18:00 to 8:00 starting Monday–Thursday |
|  | Weekend | Friday 18:00 to Monday 8:00 |

## Table S4

Grouped nonspecific discharge diagnoses and frenquency of underlying primary diagnoses.

| Nonspecific discharge diagnosis group  According to grouping by Gregersen et al. (REF) | | Frequency |
| --- | --- | --- |
| **Headache** | R519 Headache, unspecified | 3049 |
| **Observation for stroke** | Z033E Obs. pga mistanke om apopleksi | 771 |
| **Fainting** | R559 Fainting or collapse | 2906 |
|  | R559A Vasovagal syncope | 1204 |
|  | R559B Syncope, unspecified | 1669 |
|  | R559D Collapse, unspecified | 54 |
| **Observation for nervous system disorder, unspecified** | Z033 Observation for suspected nervous system disorder | 1074 |
| **Seizures or muscle cramps** | R252 Cramps or spasms | 65 |
|  | R252A Cramps, unspecified | 983 |
|  | R252B Spasms, unspecified | <10 |
|  | R568 Other or unspecified convulsions | 72 |
|  | R568C Emotional (affective) seizures | <10 |
|  | R568D Seizures, unspecified | 381 |
|  | R568E First-time unprovoked generalized TCS (GTCS) | <10 |
|  | R568G Non-epileptic seizures | 30 |
| **Symptoms involving the nervous and musculoskeletal system** | R200 Loss of sensation in skin | 12 |
|  | R201 Reduced sensation in skin | 31 |
|  | R202 Abnormal sensation in skin | 67 |
|  | R203 Hyperesthesia in skin | <10 |
|  | R208 Other or unspecified disorders of skin sensation | 230 |
|  | R251 Tremor, unspecified | 41 |
|  | R252C Muscle spasms, unspecified | 33 |
|  | R253 Fasciculations | <10 |
|  | R258 Other or unspecified abnormal involuntary movements | <10 |
|  | R258A Myoclonus | <10 |
|  | R262 Difficulty in walking, not elsewhere classified | 11 |
|  | R268 Other specified difficulties in walking and mobility | <10 |
|  | R268C Other difficulties in walking | <10 |
|  | R268D Uncertainty about legs, unspecified | 15 |
|  | R268F Difficulty in walking, unspecified | 38 |
|  | R270 Ataxia, unspecified | <10 |
|  | R278 Other and unspecified coordination disorders | <10 |
|  | R278A Coordination disorder, unspecified | <10 |
|  | R290A Carpopedal spasm | <10 |
|  | R294 Hip click | <10 |
|  | R298 Other and unspecified symptoms and signs involving the nervous and musculoskeletal systems | 381 |
|  | R298A Symptom of nervous system, unspecified | 1791 |
|  | R470 Dysphasia or aphasia | <10 |
|  | R470A Dysphasia | <10 |
|  | R470B Aphasia | 16 |
|  | R471 Dysarthria or anarthria | <10 |
|  | R471A Dysarthria | 10 |
|  | R478 Other or unspecified speech disturbance | 10 |
|  | R482 Apraxia | <10 |
| **Vertigo** | R200 Loss of sensation in skin | 3693 |
| **Observation for concussion** | Z033D Observation for concussion | 1742 |
| **Epistaxis and oropharyngeal bleeding** | R040 Epistaxis | 1016 |
|  | R040A Epistaxis anterior | 247 |
|  | R040B Epistaxis posterior | 131 |
|  | R040C Epistaxis recurring | 66 |
|  | R041 Pharyngeal bleeding | 23 |
| **Chest pain** | R071 Chest pain on breathing | 741 |
|  | R072 Precordial pain | <10 |
|  | R073 Other chest pain | 1696 |
|  | R074 Chest pain, unspecified | 9073 |
| **Observation for myocardial infarction** | Z034 Observation for suspected myocardial infarction | 3095 |
| **Palpitations** | R002 Palpitations | 1484 |
| **Abnormal heart rhythm** | R000 Tachycardia, unspecified | 186 |
|  | R001 Bradycardia, unspecified | 263 |
|  | R008 Other and unspecified abnormalities of heart beat | <10 |
|  | R008A Cardiac arrhythmia, unspecified | 54 |
| **Observation for arrhythmia** | Observation for suspected arrhythmia | 548 |
| **Observation for other cardiovascular disease** | Z035 Observation for other suspected cardiovascular diseases | 1819 |
|  | Z035C Observation for suspected stable angina pectoris | 172 |
|  | Z035D Observation for suspected valvular disease or endocarditis | 24 |
|  | Z035E Observation for suspected heart failure | 87 |
| **Coughing** | R059 Cough, unspecified | 857 |
|  | R093 Abnormal sputum | <10 |
| **Abnormal breathing** | R060 Dyspnoea | 5015 |
|  | R060A Orthopnea | 69 |
|  | R061 Stridor | 21 |
|  | R064 Hyperventilation | 155 |
|  | R068 Other and unspecified abnormalities of breathing | 29 |
|  | R068C Choking sensation | <10 |
|  | R068D Apnea, unspecified | <10 |
|  | R090 Asphyxia | <10 |
| **Abdominal pain** | R100 Acute abdominal pain | 8866 |
|  | R100A Guarding in acute abdomen | <10 |
|  | R101 Pain localized to upper abdomen | 2336 |
|  | R102 Pelvic and perineal pain | 133 |
|  | R102A Abdominal pain localized to perineum | 10 |
|  | R102B Abdominal pain localized to pelvis | 28 |
|  | R102C Abdominal pain localized to lower abdomen | 1847 |
|  | R103 Abdominal pain localized to other parts of lower abdomen | 394 |
|  | R103A Defecation pain | <10 |
|  | R104 Other or unspecified abdominal pain | 2112 |
|  | R104B Abdominal colic, unspecified | 22 |
|  | R193 Guarding in abdominal wall, unspecified | <10 |
|  | R198E Rectal tenesmus | <10 |
| **Nausea and vomiting** | R119 Nausea and vomiting | 599 |
|  | R119B Nausea | 207 |
|  | R119C Vomiting | 585 |
| **Dysphagia and eating difficulties** | R139 Dysphagia unspecified | 489 |
|  | R633 Feeding difficulties and mismanagement | <10 |
|  | R633A Eating disorder, unspecified | <10 |
|  | R638 Symptoms and abnormal findings relating to food and fluid intake, unspecified | <10 |
| **Altered mental status and amnesia** | R400 Somnolence | 66 |
|  | R401 Stupor | <10 |
|  | R402 Coma, unspecified | <10 |
|  | R410  Disorientation, unspecified | 760 |
|  | R411 Anterograde amnesia | <10 |
|  | R413 Other amnesia | <10 |
|  | R413A Amnesia, unspecified | 73 |
|  | R464 Slowness and poor responsiveness | 17 |
|  | R549 Senility | <10 |
|  | R549A Senile debility | <10 |
| **Edema** | R600 Localized edema | 150 |
|  | R601 Generalized edema | <10 |
|  | R609 Edema, unspecified | 642 |
| **Fever** | R502 Drug-induced fever | <10 |
|  | R508 Other fever, unspecified | <10 |
|  | R508A Fever with chills | 32 |
|  | R508B Persistent fever | 14 |
|  | R509 Fever, unspecified | 1337 |
| **Malaise or fatigue** | R539 Malaise or fatigue, unspecified | 630 |
|  | R539A Exhaustion | 37 |
|  | R539E Feeling of illness | 54 |
|  | R539F Malaise | 2193 |
| **Tendency to fall** | R296 Tendency to fall, not elsewhere classified | 1218 |
| **Abnormal findings of the skin** | R219 Skin rash, unspecified | 299 |
|  | R220 Swelling, mass or lump in skin or subcutaneous tissue on head | 26 |
|  | R221 Swelling, mass or lump in skin or subcutaneous tissue on neck | 39 |
|  | R222 Swelling, mass or lump in skin or subcutaneous tissue on body | 41 |
|  | R223 Swelling, mass or lump in skin or subcutaneous tissue on arm | 49 |
|  | R224 Swelling, mass or lump in skin or subcutaneous tissue on leg | 213 |
|  | R227 Swelling, mass or lump in skin or subcutaneous tissue, multiple sites | <10 |
|  | R229 Swelling, mass or lump in skin or subcutaneous tissue, unspecified | 68 |
|  | R230 Cyanosis | <10 |
|  | R231 Pallor | <10 |
|  | R232 Redness | <10 |
|  | R233 Spontaneous ecchymoses | <10 |
|  | R233A Spontaneous petechiae | 11 |
|  | R234 Changes in skin texture | <10 |
|  | R238 Other specified skin changes | <10 |
|  | R238A Skin change, unspecified | 38 |
| **Symptom from urination and urinary tract** | R300 Dysuria | 49 |
|  | R301 Vesical tenesmus | <10 |
|  | R309 Pain during urination, unspecified | 28 |
|  | R319 Hematuria, unspecified | 292 |
|  | R319A Macroscopic hematuria, unspecified | 1070 |
|  | R319B Microscopic hematuria, unspecified | 55 |
|  | R329 Urinary incontinence, unspecified | 18 |
|  | R339 Urinary retention, unspecified | 1013 |
|  | R339B Acute urinary retention | 362 |
|  | R339C Chronic urinary retention | 44 |
|  | R339D Residual urine | <10 |
|  | R349B Oliguria | <10 |
|  | R359 Polyuria, unspecified | 38 |
|  | R359B Pollakiuria | 15 |
|  | R369 Discharge from urethra, unspecified | <10 |
|  | R390 Extravasation of urine | <10 |
|  | R391 Urination difficulty, unspecified | 119 |
|  | R391D Urgency of urination | <10 |
|  | R398 Other specified symptoms/abnormal findings in urinary tract | <10 |
|  | R398B Infravesical obstruction | <10 |
| **Acute pain** | R520 Acute pain | 260 |
|  | R529 Pain, unspecified | 3925 |
| **Observation for unspecified disease or condition** | Z038 Observation for other suspected diseases and conditions | 2442 |
|  | Z039 Observation for suspected disease or condition, unspecified | 4798 |
| **Abnormal biochemical result** | R700 Elevated erythrocyte sedimentation rate | <10 |
|  | R728 Other specified abnormalities of white blood cells, unspecified | <10 |
|  | R739 Hyperglycemia, unspecified | 397 |
|  | R740 Elevation of transaminases and lactate dehydrogenase | 10 |
|  | R740B Elevation of transaminases in serum | 30 |
|  | R748A Abnormal serum amylase | <10 |
|  | R748B Abnormal alkaline phosphatase concentration in serum | <10 |
|  | R768D Elevated antiphospholipid antibody titer | <10 |
|  | R770A Hypoalbuminemia, unspecified | 11 |
|  | R790 Abnormal level of minerals in blood | <10 |
|  | R790B Abnormal level of magnesium in blood | <10 |
|  | R798 Other specified abnormal findings of blood chemistry | 63 |
|  | R798E Hypoxia | 14 |
|  | R799 Abnormal chemical finding in blood, unspecified | 44 |
|  | R809 Proteinuria, unspecified | <10 |
|  | R821 Myoglobinuria | <10 |
|  | R824 Ketonuria | <10 |
|  | R827A Choluria | <10 |
|  | R845 Specimen from respiratory tract/chest with abnormal microbiological finding | <10 |
|  | R876 Specimen from female genital tract with abnormal cytological finding | <10 |
| **Observation for cancer** | Z031A Observation for suspected cancer in the central nervous system | 28 |
|  | Z031B Observation for suspected lung cancer | 152 |
|  | Z031BR Observation for suspected recurrence of lung cancer | <10 |
|  | Z031C Observation for suspected esophageal or gastric cancer | 11 |
|  | Z031C1 Observation for suspected esophageal cancer | 16 |
|  | Z031C2 Observation for suspected cardia cancer | <10 |
|  | Z031C3 Observation for suspected gastric cancer | 13 |
|  | Z031CR Observation for suspected recurrence of esophageal/gastric cancer | <10 |
|  | Z031D Observation for suspected colorectal cancer | 71 |
|  | Z031DA Observation for suspected colorectal cancer | 76 |
|  | Z031DAR Observation for suspected recurrence of colorectal cancer | <10 |
|  | Z031DB Observation for suspected rectal cancer | <10 |
|  | Z031DBR Observation for suspected recurrence of rectal cancer | <10 |
|  | Z031DC Observation for suspected anal cancer | <10 |
|  | Z031DR Observation for suspected recurrence of colorectal or rectal cancer | <10 |
|  | Z031E Observation for suspected liver cancer | 30 |
|  | Z031F Observation for suspected pancreatic cancer | 106 |
|  | Z031H Observation for suspected urinary tract cancer | 44 |
|  | Z031H1 Observation for suspected kidney cancer | 32 |
|  | Z031H1R Observation for suspected recurrence of kidney cancer | <10 |
|  | Z031H2 Observation for suspected bladder cancer | 45 |
|  | Z031H2R Observation for suspected recurrence of bladder cancer | <10 |
|  | Z031H3 Observation for suspected ureter cancer | <10 |
|  | Z031HR Observation for suspected recurrence of urinary tract cancer | <10 |
|  | Z031J Observation for suspected prostate cancer | 27 |
|  | Z031K Observation for suspected female genital cancer | <10 |
|  | Z031K1 Observation for suspected ovarian cancer | 47 |
|  | Z031K2 Observation for suspected uterine cancer | 13 |
|  | Z031K3 Observation for suspected cervical cancer | <10 |
|  | Z031K3R Observation for suspected recurrence of cervical cancer | <10 |
|  | Z031K5 Observation for suspected vulvar cancer | <10 |
|  | Z031KR Observation for suspected recurrence of female genital cancer | <10 |
|  | Z031L Observation for suspected bile duct cancer | 14 |
|  | Z031N Observation for suspected bone cancer | <10 |
|  | Z031NR Observation for suspected recurrence of bone cancer | <10 |
|  | Z031P Observation for suspected skin cancer | <10 |
|  | Z031R Observation for suspected breast cancer | <10 |
|  | Z031RR Observation for suspected recurrence of breast cancer | <10 |
|  | Z031S Observation for suspected penile cancer | <10 |
|  | Z031T Observation for suspected testicular cancer | 20 |
|  | Z031TR Observation for suspected recurrence of testicular cancer | <10 |
|  | Z031W Observation for suspected adrenal cancer | <10 |
|  | Z031X Observation for suspected head or neck cancer | <10 |
|  | Z031XD Observation for suspected thyroid cancer | <10 |
|  | Z031XE Observation for suspected laryngeal or pharyngeal cancer | <10 |
|  | Z031XF Observation for suspected lymph node cancer in the neck | <10 |
|  | Z031XFR Observation for suspected recurrence of lymph node cancer in the neck | <10 |
|  | Z031Y Observation for suspected blood or hematopoietic organ cancer | 12 |
|  | Z031YA Observation for suspected lymphoma or chronic lymphocytic leukemia | <10 |
|  | Z031YB Observation for suspected chronic myeloid disease | <10 |
|  | Z031YC Observation for suspected acute leukemia | <10 |
|  | Z031YD Observation for suspected myeloma | <10 |
|  | Z038E Observation for suspected tumor, unspecified | 14 |
| **Other unspecific diagnoses** | R011 Heart murmur, unspecified | 14 |
|  | R030 Elevated blood pressure without documented hypertension | 54 |
|  | R030A Transient elevation of blood pressure | 27 |
|  | R030B White coat hypertension | <10 |
|  | R031 Low blood pressure, unspecified | 21 |
|  | R042 Hemoptysis | 272 |
|  | R048 Bleeding from other specified sites in respiratory tract | <10 |
|  | R048A Bleeding from lungs | <10 |
|  | R049 Bleeding from respiratory tract, unspecified | <10 |
|  | R065A Snoring | <10 |
|  | R066 Hiccup | 17 |
|  | R070 Throat pain | 55 |
|  | R098 Other specified symptoms and signs concerning the circulatory and respiratory systems | <10 |
|  | R098B Crepitation on lung auscultation | <10 |
|  | R129 Heartburn, unspecified | 40 |
|  | R131 Sleep-related swallowing, coughing, or laryngospasm | <10 |
|  | R131A Sleep-related swallowing difficulty | <10 |
|  | R149 Flatulence or similar condition, unspecified | <10 |
|  | R159 Fecal incontinence | 15 |
|  | R160 Enlarged liver, unspecified | <10 |
|  | R161A Splenomegaly, unspecified | <10 |
|  | R162 Hepatomegaly with splenomegaly, unspecified | <10 |
|  | R179 Jaundice, unspecified | 167 |
|  | R189 Ascites, unspecified | 248 |
|  | R190 Abdominal or pelvic swelling, unspecified | <10 |
|  | R190B Umbilical swelling, unspecified | <10 |
|  | R190C Abdominal swelling, unspecified | <10 |
|  | R194 Altered bowel habit | 87 |
|  | R195 Abnormal feces, unspecified | 85 |
|  | R196 Halitosis | <10 |
|  | R198 Other specified symptoms and signs concerning the digestive system and abdomen | <10 |
|  | R198A Belching | <10 |
|  | R198C Meteorism | <10 |
|  | R198G Burning mouth syndrome | <10 |
|  | R418 Other specified symptoms and signs concerning cognition | <10 |
|  | R438B Disturbance of taste, unspecified | <10 |
|  | R438C Disturbance of smell, unspecified | <10 |
|  | R440 Auditory hallucinations | <10 |
|  | R441 Visual hallucinations | 12 |
|  | R442 Other hallucinations | <10 |
|  | R443 Hallucinations, unspecified | 15 |
|  | R448 Other specified symptoms and signs concerning perception | <10 |
|  | R450 Nervousness | <10 |
|  | R451 Restlessness or agitation | <10 |
|  | R451A Restlessness | <10 |
|  | R451B Agitation | 24 |
|  | R452 Unhappiness or worry | <10 |
|  | R452B Worry | 80 |
|  | R454 Irritability or anger | <10 |
|  | R458 Other specified symptoms and signs concerning mood | <10 |
|  | R462 Odd or unexplained behavior | <10 |
|  | R466 Excessive worrying or preoccupation with distressing experiences | 14 |
|  | R490A Hoarseness | 14 |
|  | R498B Change in voice, unspecified | <10 |
|  | R521 Malignant pain | 29 |
|  | R521A Simple malignant pain | <10 |
|  | R521B Complex malignant pain | <10 |
|  | R522 Other chronic pain | <10 |
|  | R522A Chronic pain, unspecified | 40 |
|  | R522B Pain in genitals | 95 |
|  | R522D Simple long-standing or chronic non-malignant pain | 18 |
|  | R522E Complex long-standing or chronic non-malignant pain | 71 |
|  | R589 Bleeding, unspecified | 230 |
|  | R590 Localized lymphadenopathy | 10 |
|  | R599 Lymphadenopathy, unspecified | 31 |
|  | R610 Localized hyperhidrosis | <10 |
|  | R619 Hyperhidrosis, unspecified | <10 |
|  | R619A Night sweats | <10 |
|  | R630 Loss of appetite | 17 |
|  | R631 Polydipsia | <10 |
|  | R634 Abnormal weight loss | 95 |
|  | R635 Abnormal weight gain | <10 |
|  | R649 Cachexia, unspecified | 12 |
|  | R679 Generalized decrease in functional capacity, unspecified | 47 |
|  | R680 Hypothermia, not due to cold environment | <10 |
|  | R682 Dry mouth, unspecified | <10 |
|  | R688 Other general symptoms and signs | <10 |
|  | R688A Medically unexplained symptom | <10 |
|  | R688A9 Functional disorder, unspecified | <10 |
|  | R688A9B1 Functional disorder, general/fatigue | 17 |
|  | R688A9B3 Functional disorder, musculoskeletal | <10 |
|  | R688A9B5 Functional disorder, neurological | 11 |
|  | R688A9C Functional disorder, single symptom | <10 |
|  | R699 Illness, unspecified | <10 |
|  | R699A | <10 |
|  | R780 Alcohol in blood | 271 |
|  | R781 Morphine in blood | <10 |
|  | R782 Cocaine in blood | <10 |
|  | R784 Other drugs in blood | <10 |
|  | R785 Psychoactive drugs in blood | <10 |
|  | R788A Increased lithium level in blood | 11 |
|  | R900 Intracranial space-occupying lesion | <10 |
|  | R919 Abnormal imaging findings in lung | 24 |
|  | R931 Abnormal imaging findings in heart/coronary vessels | <10 |
|  | R932 Abnormal imaging findings in liver and biliary tract | <10 |
|  | R938 Abnormal imaging findings in other specified structures | <10 |
|  | R939 Abnormal imaging findings, unspecified | <10 |
|  | R941 Abnormal function study of peripheral nervous system/sensory organ | <10 |
|  | R944 Abnormal kidney function study | <10 |
|  | R945 Abnormal liver function study | <10 |
|  | R946 Abnormal thyroid function study | <10 |
|  | Z030 Observation for suspected tuberculosis | 18 |
|  | Z032 Observation for suspected mental disorder or behavioral disturbance | 10 |
|  | Z033A Observation for suspected epilepsy | 133 |
|  | Z033B Observation for suspected migraine | 22 |
|  | Z033C Observation for suspected disseminated sclerosis | 12 |
|  | Z033F Observation for suspected consequence of brain injury | <10 |
|  | Z036 Observation for suspected toxic effect of ingested substance | 27 |
|  | Z038A Observation for suspected urinary tract infection | 268 |
|  | Z038B Observation for suspected urinary tract disease, unspecified | 37 |
|  | Z038C Observation for suspected allergic disorder | 118 |
|  | Z038D Observation for suspected blood disorder | 38 |
|  | Z038F Observation for suspected malnutrition | <10 |
|  | Z038G Observation for suspected physical abuse | <10 |
|  | Z038H Observation for suspected rheumatic or connective tissue disease | 31 |
|  | Z038J Observation for suspected smoke inhalation | 175 |
|  | Z038K Observation for suspected gastrointestinal disease | 24 |
|  | Z038KA Observation for suspected gastrointestinal perforation | <10 |

##

## Table S5

Grouped nonspecific discharge diagnoses and frenquency of underlying primary diagnoses.

| *Cluster* | *Readmission* | *Mortality* |
| --- | --- | --- |
| 1 | 4.7% (3.9-5.7%) | Censored, few events |
| 2 | 5.2% (4.4-6.2%) | Censored, few events |
| 3 | 14% (12-15%) | 2.2% (1.7-2.9%) |
| 4 | 16% (14-19%) | 3.8% (2.9-4.8%) |
| 5 | 7.1% (6.0-8.4%) | 0.5% (0.2-0.9%) |
| 6 | 6.5% (6.0-7.1%) | Censored, few events |
| 7 | 6.0% (5.4-6.6%) | Censored, few events |
| 8 | 4.5% (4.0-5.1%) | Censored, few events |
| 9 | 16% (14-18%) | 0.7% (0.4-1.2%) |
| 10 | 15% (14-17%) | 2.5% (2.0-3.2%) |
| 11 | 11% (10-12%) | 1.5% (1.2-1.8%) |
| 12 | 17% (15-19%) | 4.9% (3.8-6.3%) |
| 13 | 11% (9.4-14%) | 0.9% (0.4-1.7%) |
| 14 | 22% (20-25%) | 6.4% (4.9-8.5%) |
| 15 | 16% (13-19%) | 3.0% (2.0-4.4%) |
| 16 | 19% (17-21%) | 1.8% (1.2-2.8%) |
| 17 | 16% (13-19%) | 1.5% (0.8-2.8%) |
| 18 | 18% (15-22%) | 4.8% (3.3-6.8%) |
| 19 | 28% (25-31%) | 9.0% (7.1-11.5%) |
| 20 | 28% (24-33%) | 2.1% (1.0-4.2%) |

# References

1. Huang Z. Extensions to the k-Means Algorithm for Clustering Large Data Sets with Categorical Values. *Data Min Knowl Discov*. 1998;2:284-290. doi:10.3923/ajbmb.2011.284.290

2. Cao F, Liang J, Bai L. A new initialization method for categorical data clustering. *Expert Systems with Applications*. 2009;36(7):10223-10228. doi:10.1016/j.eswa.2009.01.060

3. Rousseeuw PJ. Silhouettes: A graphical aid to the interpretation and validation of cluster analysis. *Journal of Computational and Applied Mathematics*. 1987;20(C):53-65. doi:10.1016/0377-0427(87)90125-7

4. Zhang Z, Murtagh F, Poucke S Van, Lin S, Lan P. Hierarchical cluster analysis in clinical research with heterogeneous study population : highlighting its visualization with R. 2017;5(4). doi:10.21037/atm.2017.02.05

5. Ward JH. Hierarchical Grouping to Optimize an Objective Function. *Journal of the American Statistical Association*. 1963;58(301):236-244. doi:10.1080/01621459.1963.10500845
